# Prediction of Antibiotic Resistance Genes in Cyanobacterial Strains by Whole Genome Sequencing

**DOI:** 10.3390/microorganisms13061252

**Published:** 2025-05-28

**Authors:** Duarte Balata, Tânia Rosado, Francisco Pina-Martins, Vera Manageiro, Carina Menezes, Eugénia Ferreira, Octávio S. Paulo, Manuela Caniça, Elsa Dias

**Affiliations:** 1 Centre for Ecology, Evolution and Environmental Changes (CE3C) & CHANGE—Global Change and Sustainability Institute, Faculty of Sciences, University of Lisbon, 1749-016 Lisbon, Portugal; duarte.balata@hotmail.com (D.B.); fpinamartins@gmail.com (F.P.-M.); octavio.paulo@fc.ul.pt (O.S.P.); 2 Laboratory of Biology and Ecotoxicology, Department of Environmental Health, National Institute of Health Dr. Ricardo Jorge (INSA), 1649-016 Lisbon, Portugal; tania.s.rosado@gmail.com (T.R.); carina.menezes@insa.min-saude.pt (C.M.); 3 National Reference Laboratory of Antibiotic Resistance and Healthcare Associated Infections, Department of Infectious Diseases, National Institute of Health Dr. Ricardo Jorge, 1649-016 Lisbon, Portugal; vera.manageiro@insa.min-saude.pt (V.M.); eugenianunesferreira@gmail.com (E.F.); manuela.canica@insa.min-saude.pt (M.C.); 4 Centre for the Studies of Animal Science (CECA), Institute of Agrarian and Agri-Food Sciences and Technologies, University of Porto, 4099-002 Porto, Portugal; 5 Associate Laboratory for Animal and Veterinary Sciences (AL4AnimalS), 1300-477 Lisbon, Portugal

**Keywords:** antibiotic resistance, aquatic resistome, freshwater bacteria, cyanobacteria, genome, bioinformatics pipeline

## Abstract

Cyanobacteria are ubiquitous in freshwater environments, but their role in aquatic resistome remains unclear. In this work, we performed whole genome sequencing on 43 cyanobacterial strains isolated from Portuguese fresh/wastewaters. From 43 available non-axenic unicyanoabacterial cultures (containing only one cyanobacterial strain and their co-occurring bacteria), it was possible to recover 41 cyanobacterial genomes from the genomic assemblies using a genome binning software, 26 of which were classified as high-quality based on completeness, contamination, N50 and contig number thresholds. By using the comprehensive antibiotic resistance database (CARD) on the assembled samples, we detected four antibiotic resistance gene (ARG) variants, conferring resistance in pathogenic bacteria to tetracyclines, fluoroquinolones (*adeF*-type) and macrolides (*ermF*-type, *mefC*-type and *mphG*-type). Among these, *adeF*-type was the most prevalent gene, found across 11 cyanobacterial genomes from the Nostocales order. *Planktothrix* presented the highest variety of close ARG matches, with hits for the macrolide resistance genes *ermF*-type, *mefC*-type and *mphG-*type. An analysis of the genomic assemblies also revealed an additional 12 ARGs in bacteria from the phyla Firmicutes, Proteobacteria and Bacteroidetes, present in the cyanobacterial cultures, foreseeing the horizontal gene transfer of ARGs with cyanobacteria. Additionally, more than 200 partial ARGs were detected on each recovered cyanobacterial genome, allowing for future studies of antibiotic resistance genotype/phenotype in cyanobacteria. These findings highlight the importance of further efforts to understand the role of cyanobacteria on the aquatic resistome from a One Health perspective.

## 1. Introduction

In recent decades, due to the overconsumption and misuse of antibiotics, as well as the lack of new available antibiotics, bacteria’s resistances are starting to catch up on the substances available to control their nefarious impacts [1]. Antibiotic resistance (AR) is one of the biggest threats to global health, and it is rising dangerously [2] and driving us to a post-antibiotic era, that is, to the resistance era [3,4].

AR should not be considered only from the clinical perspective, as there is a risk of not seeing the “whole picture”. It has been argued that the discussion about AR must include evolutionary and ecological perspectives given the role of environmental microbes as sources and dissemination vehicles of AR [4]. Water compartments, particularly, are important reservoirs of resistant microorganisms (both native and pathogenic bacteria) as well as pools of antibiotic resistance genes (ARGs) [5,6,7].

Even though it is recognized, the aquatic resistome is still under investigation, namely some bacterial groups that might play an important role as reservoirs of ARGs, such as cyanobacteria [8]. Indeed, in previous studies, we showed that non-axenic cyanobacterial strains from distinct genera exhibit a non-susceptible phenotype to antibiotics that are common in freshwater, such as trimethoprim and nalidixic acid [9,10]. Additionally, we identified a class-1-type integron, *sul1*, *strA*-*strB* and *qacΔE* genes in fresh- and wastewater cyanobacterial strains (non-axenic), supporting the hypothesis that cyanobacteria can acquire and transfer AR determinants [10]. In these studies, ARGs were detected through PCR amplification, using primers and PCR conditions designed for bacteria, considering that no specific PCR conditions and primers were established for cyanobacteria. Thus, it remained unclear whether these genes were from cyanobacteria and/or whether they belonged to their associated bacteria. Indeed, the isolation and purification of cyanobacteria in cultures is a difficult task, and most strains in culture collections are non-axenic, containing associated heterotrophic bacteria, similarly to what happens in the environment [11]. In fact, the importance of these bacteria for the long-term culturing of cyanobacteria is a matter of discussion.

Other recent studies have shown that cyanobacterial blooms may influence the composition of the antibiotic-resistant bacterial community in freshwater reservoirs, thus indirectly affecting the ARG profiles in these environments [12,13]. In addition, it was also demonstrated that cyanobacteria-associated eDNA carrying ARGs may be relevant for the acquisition and dissemination of ARGs in aquatic environments [14]. The same authors [15] also used qPCR to detect genes conferring resistance to major classes of antibiotics commonly administrated to humans and animals, namely sulfonamides (*sul1*, *sul2*), tetracycline (*tetA*, *tetB*) and quinolones (*qnrB*), in bacteria-free cyanobacterial strains from eutrophic lakes in China. The results from these studies strongly support the hypothesis that cyanobacterial blooms may play a role in the aquatic resistome. However, it still remains to be demonstrated whether the ARGs present in cyanobacterial blooms are indeed present in cyanobacterial genomes. Meanwhile, the distribution and diversity of ARGs in 862 publicly available cyanobacterial genomes (up to 2021) was reported in 2023 [16]. These authors found distinct ARGs in 10% of these genomes, providing another clue to clarify the role of aquatic and terrestrial cyanobacteria in the environmental resistome.

Cyanobacteria have been found to display highly plastic and variable genomes due to the process of horizontal gene transfer (HGT) [17]. This process is common in cyanobacteria and plays an important role in their evolution [18]. HGT allows bacteria to acquire exogenous genes from mobile genetic elements (MGEs) that are not present in their clonal lineage. It is therefore plausible to suppose that HGT can facilitate the transfer of antibiotic resistance coding sequences between cyanobacteria cells and pathogenic bacteria present in the freshwater bodies, where cyanobacteria are highly abundant. The persistent ARGs found in the environment are currently considered as emerging environmental pollutants and the increased morbidity associated with their dissemination has become a global concern for public health [19].

Despite this, cyanobacteria are a highly understudied group of microorganisms in what concerns their antibiotic resistance profile. This is probably because cyanobacteria are considered non-pathogenic to humans and difficult to isolate into pure cultures.

In the continuity of our above-mentioned studies [9,10], here, we describe a genome-sequencing approach to predict ARGs in 43 cyanobacteria strains that were previously evaluated for their antibiotic susceptibility phenotype and, for some of them, their antibiotic-resistant genotype. These strains belong to species of distinct orders, namely Chroococcales (e.g., *Microcystis aeruginosa*), Nostocales (e.g., *Anabaena* spp., *Aphanizomenon* spp.) and Oscillatoriales (e.g., *Planktothrix* spp.), which are highly abundant in Portuguese freshwater environments. These strains are deposited in the ESSACC [20] and LEGE-CC ([21,22]; https://lege.ciimar.up.pt/, accessed on 21 May 2025) culture collections and were previously isolated from surface freshwater and from a wastewater treatment plant, respectively.

This study’s goal is to improve the understanding of the importance of cyanobacteria as potential ARG reservoirs in freshwater environments through the whole genome sequencing (WGS) of non-axenic/unicyanobacterial cultures of freshwater cyanobacteria from different species. We developed a pipeline for this type of approach that is easily scalable and reproducible, as well as user-friendly, in order to be implemented and expanded in future analysis.

## 2. Materials and Methods

### 2.1. Cyanobacterial Strains

Forty-three cyanobacterial strains previously isolated from Portuguese surface freshwaters and from a wastewater treatment plant (WWTP) were studied (Supplementary Figure S1). Among these strains, 31 were isolated from 20 surface freshwater reservoirs, 4 from 2 rivers and 8 from a WWTP secondary decanter tank. The freshwater strains were isolated between 1996 and 2015, being maintained since then at the “Estela Sousa e Silva Algae Culture Collection” (Laboratory of Biology and Ecotoxicology, National Institute of Health_INSA, Lisbon, Portugal) [20]. The wastewater strains were isolated between 2006 and 2007 and belong to the “LEGE Culture Collection” (Laboratory of Ecotoxicology, Genomics and Evolution, Interdisciplinary Centre of Marine and Environmental Research_CIIMAR, Porto, Portugal) [21,22] (https://lege.ciimar.up.pt/, accessed on 21 May 2025).

Non-axenic/unicyanobacterial cultures of these strains were successfully maintained in laboratory culture conditions, namely in Z8 liquid medium [23] under a 14/10 h or 12 h/12 h light/dark cycle (L/D; light intensity 16 ± 4 μEm^−2^ s^−1^, approx.) at 20 ± 1 °C [10]. Cultures of *Anabaena* spp. (9 strains), *Aphanizomenon* spp. (9 strains), *Microcystis* spp. (9 strains) and *Planktothrix* spp. (16 strains) were prepared for further DNA extraction. The taxonomy of these strains was previously determined (at the isolation date) by microscopy according to the morphometric features and identification keys described in EN 15204:2006 [24] (Supplementary Table S1). As explained in the Section 3, some of the species were re-identified after analyzing the sequencing data obtained in the present work and confirming their morphological features. In addition, reclassification also took into account the revisions that were carried out by taxonomists for some species/genera.

### 2.2. DNA Extraction and Whole Genome Sequencing

Genomic DNA was extracted from 15 mL of cultures of the above-mentioned 43 cyanobacterial strains. Cells were harvested by centrifugation (SORVALL RC-5C Plus, Kendro Laboratory Products, Asheville, NC, USA) at 4000× *g* for 25 min, and DNA was extracted using a PowerWater^®^ DNA Isolation Kit (MoBio, Carlsbad, CA, USA) according to the manufacturer’s instructions. The DNA samples were analyzed by gel electrophoresis (0.8% agarose, *w*/*v*) and quantified using a Qubit^®^ 3.0 fluorometer (Thermo Fisher Scientific, Waltham, MA, USA).

Sequencing libraries were prepared with a Nextera XT DNA Sample Preparation Kit (Nextera XT DNA Library Prep Kit Reference Guide 15031942v03, February 2018, Illumina) according to the manufacturer’s instructions. Briefly, the genomic DNA extracted from each cyanobacteria culture (0.2 ng/µL) was tagmented at 55 °C for 5 min. Illumina index sequences (Nextera XT Index Kit, FC-131-1001, Illumina, San Diego, CA, USA) were added to each tagmented DNA sample by PCR.

The library DNA fragments were size-selected and purified using AMPure XP beads (Beckman Coulter, Inc.), and the quality control, including the size and concentration of each library, was estimated on a Fragment Analyzer (HS NGS Fragment Kit 1-6000 bp, DNF-474, Agilent, Santa Clara, CA, USA).

Libraries were normalized and pooled to 4 nM. Pooled libraries were denatured and diluted (Denature and Dilute Libraries Guide, Document # 15,039,740 v10, February 2019, Illumina) to a final concentration of 15 pM/20 pM (V2/V3 Illumina sequencing kit (MS-103-1003/MS-102-3003), respectively) with 1% of Phix (FC-110-3001, Illumina) as sequencing control. Whole genome sequencing was carried out using a 2 × 250 paired-end (PE) reads configuration on a high-throughput Illumina MiSeq platform in the Technology and Innovation Unit—Department of Human Genetics (UTI-DGH) facilities from INSA. Image analysis and base calling were conducted by MiSeq Control Software (MCS) directly on the MiSeq instrument (Illumina, San Diego, CA, USA).

The use of non-axenic/unicyanobacterial cultures means that each cyanobacteria culture contains not only a single cyanobacterial strain but also bacteria that were present in the water samples from which the cyanobacteria were isolated and that were co-cultured with the cyanobacterial strain. In this sense, the total DNA obtained from each cyanobacteria culture corresponds, indeed, to a metagenome. In this work, we aimed to recover cyanobacterial genomes from the correspondent metagenomes, and therefore, these are henceforth called “genomes” and not “metagenomes”.

### 2.3. Data Analysis

#### 2.3.1. Sequence Filtering

The raw Illumina MiSeq output files in fastq format were analyzed using FastQC version 0.11.8. This software provided insights on read quality scores, per-base sequence content and per-sequence GC content. Anomalous base content regions in the beginning and trailing portion of the reads, resulting from the presence of Illumina sequencing adapters, were trimmed utilizing Cutadapt version 1.18.

Reads were also filtered by quality score using BBTools suite’s BBDuk version 38.32. Every read with an average Phred quality score below 33 was discarded from the analysis. BBDuk was also used to create interleaved fastq files from the original Illumina paired read format file, which were needed in downstream software.

#### 2.3.2. Taxonomy Classification

Sequences were taxonomically classified using Kraken2 [25] version 2.0.7 and the SILVA 16S database [26], which, at the time of this work, presented information on a higher variety of cyanobacteria genera than the Refseq genomic database.

#### 2.3.3. Genome Assembly and Genome Binning

The reads were assembled with SPAdes [27] version 3.13.1, using careful mode with kmer lengths of 21, 33, 55, 77, 99 and 127.

The assembled genome was separated into bins corresponding to the multiple species present in the communities, using MaxBin2 [28] version 2.2.6. This software was chosen because it can bin contigs as short as 500 bp, being therefore suited for lower coverage/more fragmented genomes. Using this genomic binning software, each genome was binned into 2 to 12 genomic bins, each corresponding to a taxonomic group, based on their sequencing depth and GC base content. Since this work’s main goal was to recover cyanobacteria genomes, the bins containing cyanobacteria sequences were identified amongst the remaining bacteria by comparing their genome characteristics (length, GC%) to reference genomes of the same genera, as well as querying the obtained sequences against reference databases using BLAST [29] version 2.11.0.

An in-house Python script named pick_bin.py (commit ‘b36d096’) was made to access the summary files produced by MaxBin2 and automatically select the bin that is most similar to the provided cyanobacterial reference. This selection was performed based on a comparison of the weighted arithmetic means of GC percentage and genome size between the bin and reference genome. A weight of 90% was attributed to the GC content parameter since it works as an identifier for the genera and is less subject to being compromised by the lack of data for some genomic regions. The remaining 10% was attributed to the length of the genome since it is considerably more variable than GC% and highly subject to bias when the whole genome is present for sequencing reasons. Despite this, this parameter is important, mostly to discard very small bins (likely to be binning artifacts) or when dealing with species with close GC contents.

The bins picked by pick_bin.py were analyzed with QUAST [30] version 5.0.2 in order to create a genome assembly quality report. This software presented many insightful assembly statistics, such as genome length, N50, GC content and number of contigs per assembly. N50 measures assembly quality in terms of contiguity and is defined as the sequence length of the shortest contig at 50% of the total genome length.

The multiple generated bins were then classified using Kraken2 again to attest for pick_bin.py’s bin selection and to obtain a sense of the taxonomy of the non-cyanobacterial bins. In all instances of Kraken2′s use, the Pavian online tool version 1.0 was used to obtain an interactive table visualization of the attributed taxonomy labels and well as Sankey plots.

Some of the reads were then mapped to their corresponding SPAdes assembled genome using BWA [31] version 0.7.12, and the resulting alignment SAM files were sorted and indexed using Samtools [32] version 1.9. These SAM alignments were visualized using Tablet [33] version 1.19.05.28 in order to explore the contig regions of previously PCR-amplified genes from our strains and confirm the effectiveness of the utilized SPAdes assembling parameters by checking the contiguity of previously known sequences obtained from the PCR sequencing of our samples.

CheckM [34] version 1.1.3 was used to analyze the binned genomes and calculate their estimated levels of completeness and contamination.

Raw sequence reads used for the assembly were deposited in the National Center for Biotechnology Information (NCBI) Sequence Read Archive under accession numbers SAMN34233939 to SAMN34233979 (Bioproject ref. PRJNA956929).

#### 2.3.4. Antibiotic Resistance Gene Prediction

The prediction of ARGs was performed using the Resistance Gene Identifier tool, available at the CARD website [35,36], using CARD version 3.0.3 with the “Perfect, Strict and Loose hits” criteria, and it nudged >95% Loose hits to Strict. The option “High quality/coverage” was chosen for samples presenting a genomic coverage greater than 10×, while “Low quality/coverage” was used for the remaining samples.

Antibiotic resistance hits were qualified using the following CARD criteria: Perfect hits mean that a region from the query genome is exactly the same as the reference ARG, having 100% of its length and 100% alignment identity. Strict hits are sequences almost the same as that of the reference gene in either length or identity, but only partially similar in the other parameter. Proteins encoded by ‘Strict hit’ genes present a high probably of maintaining the AR trait displayed by the reference gene according to the CARD prediction algorithm. Loose hits correspond to genes that present some percentage of similarity with the ARGs from the CARD, but might not necessarily display the same function. Loose hits can help with the detection of emergent threats or distant homologs of known ARGs [36].

### 2.4. New Bioinformatics Pipeline

**Figure 1:**
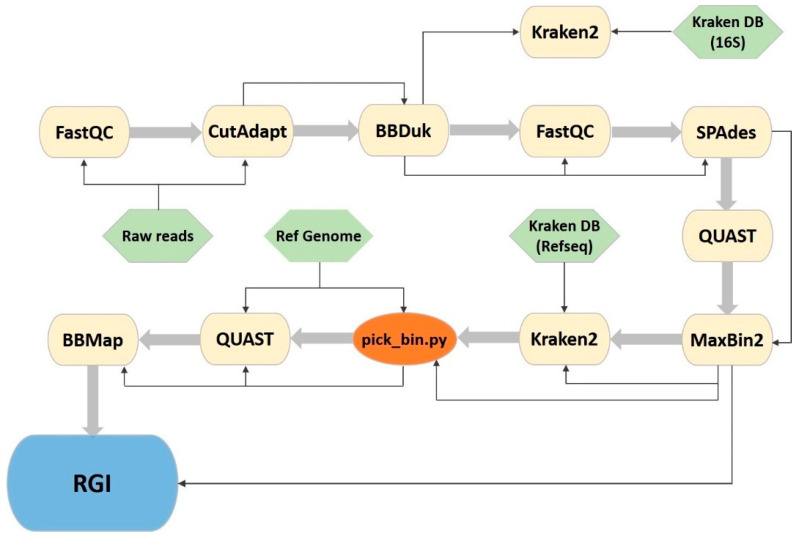
CyanoPipeline flowchart. Yellow: third party software; green: necessary pipeline input files; orange: represents a custom python script; blue: denotes a browser tool that is not included in the pipeline, but used as the final step of the analysis. Thin arrows represent the Input/Output stream and large arrows represent the succession of the software tools.

In order to automate the analysis and make it less time consuming and easily reproducible, we developed a new pipeline using GNU Make, Shell scripting and Python3. This CyanoPipeline (Figure 1; commit ‘f1cd1c4’) receives three inputs: the Illumina-produced fastq files, a reference genome of the species of interest in fasta format and a taxonomy database for Kraken2, consisting of four files (database.kdb, database.idx, taxonomy/nodes.dmp, taxonomy/names.dmp), obtained using the ‘kraken-build’ command. From these three inputs, it automatically performs all the previously described analyses (Section 2.3), giving the user the possibility to easily change some variable analysis parameters from the Makefile, namely, the read trimming length, the read quality score threshold and the number of CPU threads to use. All other parameters can also be altered easily by accessing each software’s corresponding shell scripts.

## 3. Results

### 3.1. Genome Assembly

The obtained assembly lengths for the 43 genomes ranged from 5,461,759 to 54,668,933 bp, presenting significant heterogeneity, probably due to the use of different types of flow cells during strain sequencing. As for the number of contiguous sequences (contigs) of lengths over 500 bp, generated per assembly, their quantities ranged from 1653 to 19,547. It is noticeable that samples containing *Microcystis* strains generally presented the lowest number of contigs (3565) when compared to other sample sets, presenting a mean number per sample of approximately 42% of the average global value (8460). On the other hand, *Planktothrix mougeotii* samples displayed a relatively high number of contigs when compared to others, with roughly 182% of the global average (numbers averaging 15,472 per sample).

After processing the raw reads, Kraken2 was used, along with the SILVA 16S database, to attribute taxonomic labels, at the genus level, to the reads present in each of the genomic assemblies. Using Pavian to filter the results to display only cyanobacterial matches, we obtained the results shown in Supplementary Table S2. A cyanobacterial sample was considered likely to belong to the genus to which it presents the highest matching percentage attributed by Kraken2. The matching percentage refers to the percentage of query reads that present the highest identity score with a given reference data set.

Complete metagenomic classifications of all samples are represented using Sankey diagrams in Supplementary Figure S2. Apart from the cyanobacteria, the remaining bacteria on the samples belonged primarily to the phyla Firmicutes, Proteobacteria and Bacteroidetes.

When restricting the outputs to the phylum Cyanobacteria, the *Anabaena* samples LMECYA 123C and LMECYA 161 showed the greatest correspondence with the genus *Dolichospermum* (78.58% and 85.36% of the reads, respectively). LMECYA 165, LMECYA 182 and LMECYA 204 were identified as *Sphaerospermopsis*, with 71.24%, 55.02% and 75.4% of their reads matching this genus, respectively. LMECYA 213 and LMECYA 313 matched with *Aphanizomenon* (70.14% and 23.75%, respectively), and LMECYA 246 with *Anabaena*, with 65.5% of the reads (Supplementary Table S2.1).

*Aphanizomenon* samples LMECYA 009, LMECYA 040, LMECYA 191, LMECYA 237 and LMECYA 328 were identified as belonging to the genus *Aphanizomenon*, with 37.36%, 51.12%, 48.68%, 56.65% and 73.17% of their reads matching the reference sequence, respectively. LMECYA 031 and LMECYA 190 showed the greatest correspondence with *Cuspidothrix* (48.54% and 52.04%, respectively). LMECYA 089 and LMECYA 253 were attributed to the genus *Dolichospermum*, with 46.12% and 45.29% matching (Supplementary Table S2.2).

The Kraken2 identification of all *Microcystis* samples was in conformity with the previous morphological and morphometric identification, attributing all samples to the genus *Microcystis*. These samples had between 46.84% and 69.47% of their classified reads attributed to this genus (Supplementary Table S2.3).

Every *P. agardhii* sample presented the highest matching percentage with the genus *Planktothrix*, as initially expected, except for LMECYA 303. The percentages of bases attributed to this genus were 85.92% to 90.55% (Supplementary Table S2.4).

Similarly, every *P. mougeotti* sample displayed the highest percentage of matching reads with the genus *Planktothrix*, ranging from 73.12% to 86.08% (Supplementary Table S2.5).

Considering the differences between the original taxonomic classifications of the cyanobacterial strains of *Anabaena* and *Aphanizomenon* based on morphology and the genomic data, we reclassified these strains, as presented in Supplementary Table S1. Besides the morphologic/morphometric and genomic data, we also took into account the taxonomic revisions that were carried out for the Nostocales order.

Indeed, the taxonomic identification of cyanobacteria has been historically performed through optical microscopy, according to the morphometric and morphologic features and identification keys described in EN 15204:2006. This is still a widely used method in phytoplankton and cyanobacterial bloom monitoring in freshwater reservoirs [37] and was the method used to identify the strains in this work, as referred in Section 2.1. However, ambiguity regarding several species/genera identifications and incongruencies revealed by phylogenetic information has led to taxonomic revisions in the last two decades, and polyphasic approaches, combining morphologic, molecular and ecologic criteria, have been proposed as the more adequate cyanobacteria identification procedure [38]. The order Nostocales, in particular the genera *Anabaena* and *Aphanizomenon*, has suffered many taxonomic revisions. For example, the benthic *Anabaena* morphotypes were maintained within the *Anabaena* genus, while its planktonic morphotypes were reclassified as *Dolichospermum* [39]. However, while some authors adopted the new nomenclature, others still maintained the old one for several years [40]. Another example is the *Aphanizomenon issatschenkoi* species, which was reclassified into *Cuspidothrix issatchenkoi* [41], but maintained this double nomenclature for several years [42].

In the present study, we found that some Nostocales LMECYA strains had not yet been reclassified, so the results of the genome analysis did not coincide with the initial cyanobacterial classification. This by itself is an outcome of the work, enabling the correct identification of LMECYA strains through the analysis of genomes and further confirmed by the observation of their morphological features.

### 3.2. Recovered Cyanobacterial Genomes

**Figure 2:**
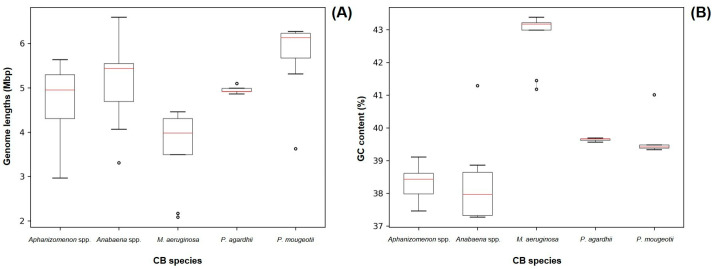
Size (**A**) and GC content (**B**) of all recovered cyanobacteria genomes grouped by morphometric taxonomy identification. Red lines represent median values. Dots represent outlier values.

Out of the 43 sequenced genomes, it was possible to recover cyanobacteria assemblies from 41 of them. The length and GC content parameters of the recovered cyanobacteria genomes are represented on Figure 2 and are close to the previously reported values for *Aphanizomenon* spp. (5.187 Mb/37.65%), *Anabaena* spp. (5.306 Mb/38.38%), *Planktothrix agardhii* (5.512 Mb/39.50%) and *Planktothrix mougeotii* (5.499 Mb/39.5%) [43,44,45,46]. For *Microcystis* spp. samples, the GC base % was close to the available *Microcystis aeruginosa* reference genome, but the lengths on our assemblies (average 3.648 Mb [±0.917 Mb]) were smaller (5.2 Mb) [47]. This can be related to the lower output of the Illumina Nano flow cell used in the sequencing of our *Microcystis* spp. strains, as it was not capable of producing reads across the entire length of the cyanobacterial genome.

**Figure 3:**
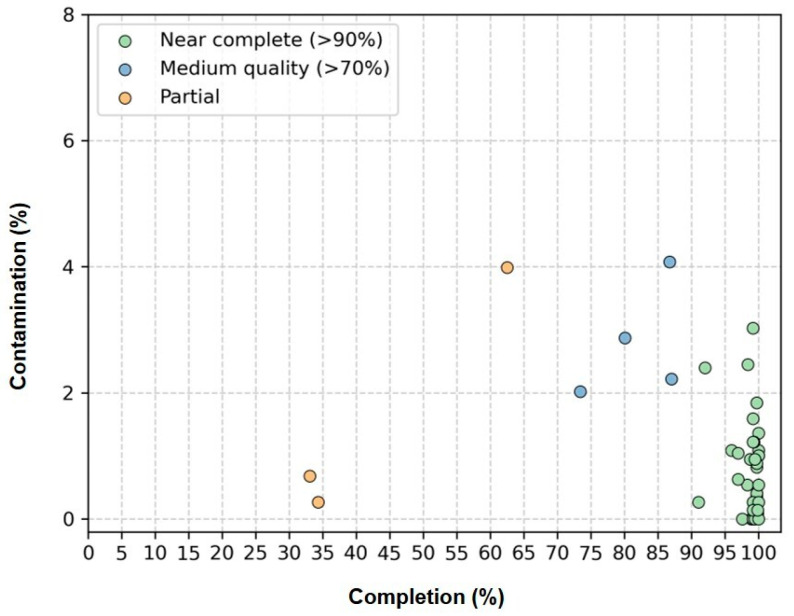
Estimated completeness and contamination of 41 cyanobacterial genomes recovered from genomes of 41 non-axenic/unicyanobacterial cultures. “Near complete” genomes (completeness ≥ 90%; contamination ≤5%) are shown in green, “Medium quality” genomes (completeness ≥70%; contamination ≤5%) in blue and “Partial” genomes (completeness ≥50%; contamination ≤5%) in yellow.

Genome quality was assessed using the CheckM software, which estimates completeness and contamination based on lineage-specific marker genes (Figure 3, Supplementary Table S3). Of the 41 cyanobacterial genomes recovered, 34 were classified as ‘Near complete’ (completeness ≥90%; contamination ≤5%); 4 were ‘Medium quality’ (completeness ≥70%; contamination ≤5%); and 3 were ‘Partial’ (completeness ≥50%; contamination ≤5%).

**Figure 4:**
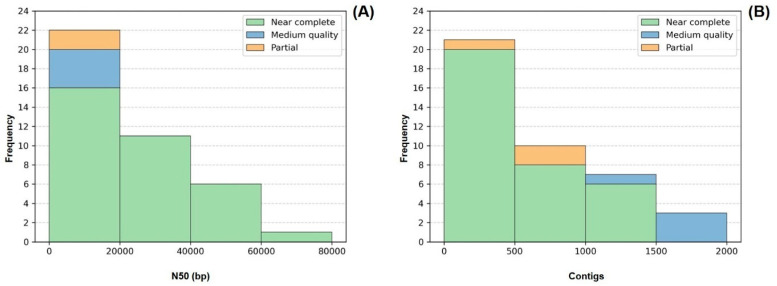
Frequency distribution of the number of contigs (**A**) and N50 values (**B**) comprising each of the 41 genomes. Colors indicate genome quality. LMECYA 089 was omitted from the histogram (**B**) since it presented an outlier value of 195,436 bp for N50.

In terms of genome fragmentation, all genomes presented fewer than 2000 contigs, and more than 50% of the recovered genomes (21/41) were composed of fewer than 500 contigs. As for N50, 26 out of the 41 recovered genomes displayed values >10 kb. The values on this parameter were heterogeneous, varying between 2 and 200 kb, which can be partly explained by the different sequencing output obtained during the sequencing step for different strains (Figure 4).

As for genome coverage, 29 out of the 41 recovered cyanobacteria genomes presented average sequencing depths above 10× (Supplementary Table S4).

Additionally, by applying the quality criteria defined by Parks et al. [48] (completeness—5× contamination > 90; N50 > 10 kb; #contigs < 1 k), we identified 26 cyanobacterial genomes as high quality. Out of the 15 lower-quality recovered genomes, 11 of them (all *Microcystis*, 1 *P. agardhii* and 1 *P. mougeotii*) could be associated with insufficient sequencing depth, which did not allow us to proceed with the ARG search.

Overall, the results confirm that most of the recovered genomes are suitable for downstream analyses, including ARG detection and taxonomic reclassification.

### 3.3. In Silico Analysis of Antibiotic Resistance Genes

All genomic bins were tested for the presence of known ARGs or close variants, including those obtained from incomplete genomes or belonging to out-target species, since they could also present valuable information about the antibiotic resistance determinants present in the bodies of water and the possible co-occurrence of similar genes between species in a community.

The in silico analysis of the assembled genomes for the presence of known ARGs showed the presence of 15 different ARGs detected using the ‘strict hit’ criteria: genes associated with antibiotic inactivation (*aadA2*-type, *aadA6/aadA10*-type, *aadA8*-type, *aadA13*-type, *aadA16*-type, *aph*(*3"*)-*Ib*-type, *aph*(*6*)-*Id*-type, *ampS*-type, *mphG*-type), with antibiotic efflux (*adeF*-type; *floR*-type, *mefC*-type, *qacH*-type, *tetD*-type) and with antibiotic target alteration (*ermF*-type). Additionally, one gene associated with target alteration was detected as a ‘perfect hit’—*sul*1 (Table 1).

**Figure 5:**
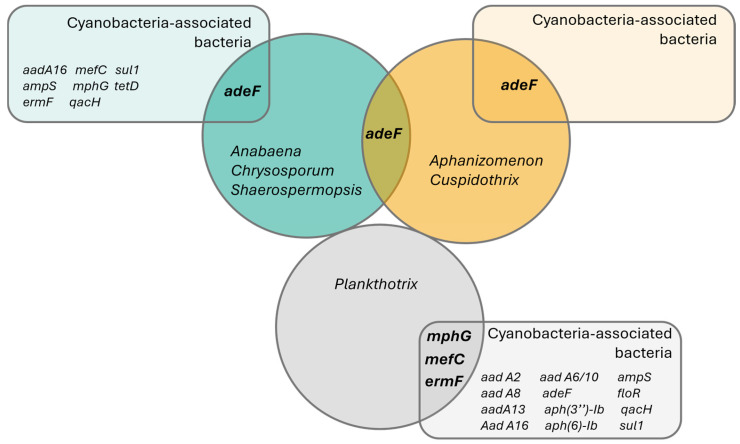
Venn diagram representing the detected antibiotic resistance genes using CARD’s ‘Perfect’ and ‘Strict’ ARG detection criteria. Circles represent sampled taxonomic groups of cyanobacteria, while the rectangles represent ARG hits detected on the co-occurring bacteria in each cyanobacterial strain culture. Interception zones represent common ARG genes in both cyanobacteria and bacteria.

From the 16 genes detected on the assembled freshwater cyanobacteria and bacteria, only 4 were present in the recovered cyanobacteria genomes—*adeF*-type, *mefC*-type, *mphG*-type and *ermF*-type (Table 1, Figure 5). All ARG hits found on the cyanobacteria genomes appeared on assemblies previously classified as ‘High quality’, according to the criteria defined by Parks et al. [48].

The *adeF*-type gene was the most widely distributed ARG in cyanobacteria, found across species from the Nostocales order (using the strains’ reclassification, Supplementary Table S1): *Anabaena torulosa* (LMECYA 313), *Chrysosporum berghii* (LMECYA 246), *Sphaerospermopsis* sp. (LMECYA 182), *Aphanizomenon* spp. (LMECYA 009, LMECYA 040, LMECYA 089, LMECYA 237, LMECYA 253, LMECYA 328) and *Cuspidothrix issatschenkoi* (LMECYA 031, LMECYA 190) on a total of 11 recovered genomes (Table 1). This gene was the only ARG detected in the Nostocales genomes, as well as the only ARG identified in co-occurring bacteria from the *Aphanizomenon* spp. and *Cuspidothrix issatschenkoi* cultures (Table 1, Figure 5).

The remaining ARGs identified in the cyanobacteria (*ermF*-type, *mphG*-type and *mefC*-type) were detected exclusively in the genus *Planktothrix* (Table 1). The *ermF*-type gene was detected on the *P. agardhii* (LMECYA 280) and *P. mougeotii* (LEGE 06224, LEGE 06226 and LEGE 07231) recovered genomes, while *mphG*-type and *mefC*-type were found exclusively on *P. mougeotii* samples (LEGE 06224 and LEGE 06226).

**Figure 6:**
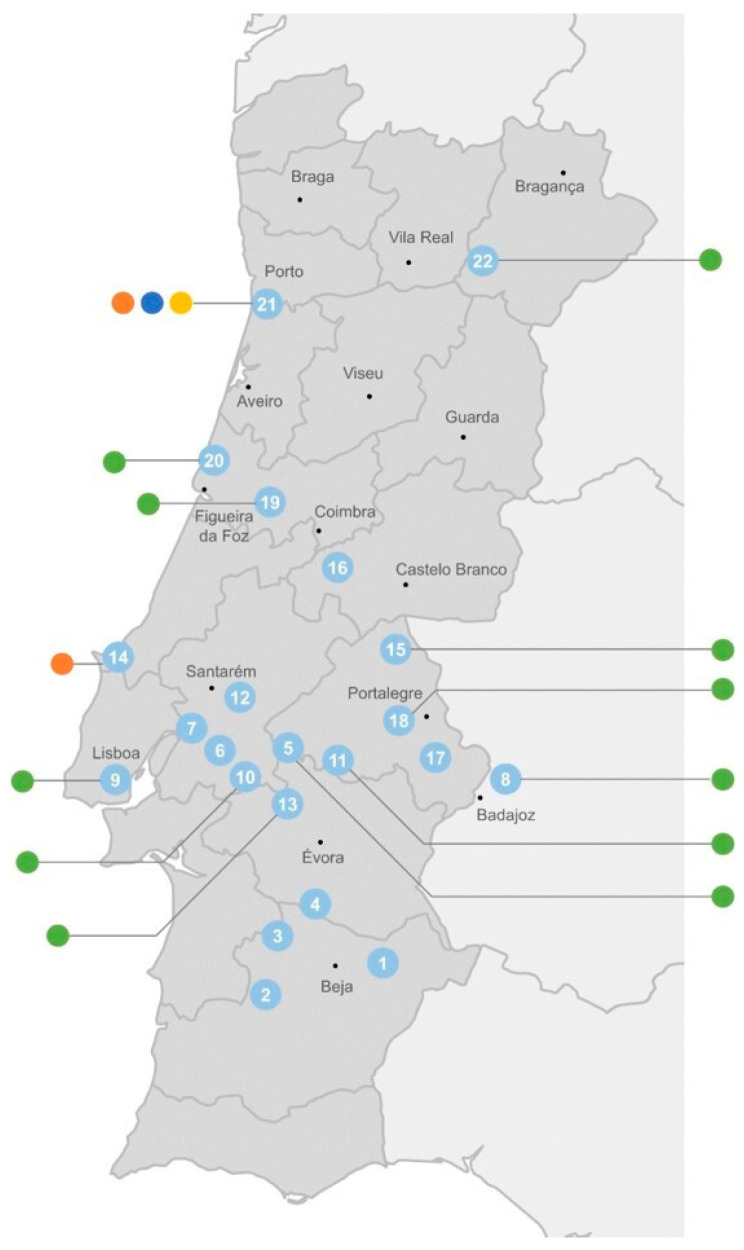
Distribution of cyanobacterial ARGs (● *adeF*-type; ● *ermF*-type; ● *mefC*-type; ● *mphG*-type) according to their sampling site (see Supplementary Figure S1 and Supplementary Table S1 for strain identification). Sampling site 21 corresponds to a wastewater treatment plant. All the other sites correspond to surface freshwater from rivers (8 and 19) and reservoirs (all remaining).

The distribution of these cyanobacterial genes according to the origin of the cyanobacterial strains shows that the *adeF*-type gene has a wide occurrence through freshwater, both from urban (mostly located on the coast) and rural areas (mostly those in the interior of the country) (Figure 6). The *ermF*-type gene was detected both in fresh- and wastewater, while the *mefC*-type and *mphG*-type genes were detected exclusively in cyanobacteria isolated from the WWTP (sampling point 21) (Figure 6).

Beyond the ARGs identified in cyanobacteria, we also detected several resistance genes exclusively in the associated bacterial genome bins, highlighting the broader resistome present in these non-axenic cultures. For example, *sul1*, a well-known sulfonamide resistance gene, was found as a perfect hit in the bacterial bins associated with the *Dolichospermum (LMECYA 161)* and *Planktothrix (LMECYA 230, LEGE 06224, LEGE 07227)* strains, showing a conserved sequence length and identity. Aminoglycoside resistance genes such as *aadA2*, *aadA13* and *aadA16* (involved in antibiotic inactivation via adenylyltransferases), as well as *aph(3’’)-Ib* and *aph(6)-Id*, were detected in bacteria co-occurring with the *Dolichospermum (LMECYA 161)*, *Planktothrix agardhii (LMECYA 230, LMECYA 280, LMECYA 283, LMECYA 292)* and *Planktothrix mougeotii (LEGE 06224, LEGE 06225, LEGE 06226, LEGE 06233, LEGE 07227, LEGE 07231)* strains. Notably, *aph(3*″*)-Ib*, *aph(6)-Id* and *floR* (a chloramphenicol/florfenicol efflux pump) were frequently co-located in bacterial genomes from *Planktothrix mougeotii* cultures, suggesting the presence of a multidrug resistance region.

## 4. Discussion

The main goal of this work was to predict the presence of ARGs in cyanobacteria isolated from freshwater and WWTP water samples to understand their putative role on the aquatic resistome using a new bioinformatic pipeline. For this purpose, non-axenic cultures of cyanobacterial strains from these environments (and whose antibiotic susceptibility phenotype we previously determined [9,10]) were analyzed by WGS to assess the presence of ARGs in the cyanobacterial genomes. The genomic data generated also allowed the discrimination of the bacteria in co-culture with the cyanobacteria under study, and consequently distinguished ARGs from both microorganisms. The genome recovery approach yielded high-quality assemblies in most cases, with completeness and contamination metrics that met the accepted standards for metagenome-assembled genomes [48], supporting the robustness of our binning strategy.

The most widely detected ARG across all samples was *adeF*-type, which was found in 25 genomic assemblies. This gene was present in 11 cyanobacterial bins, 1 belonging to *Anabaena torulosa, Chrysosporum bergii* and *Sphaerospermopsis* sp., 6 to *Aphanizomenon* spp. and 2 to *Cuspidothrix issastschenkoi*. It was also the only ARG detected on the genomes of cyanobacteria from the Nostocales order, as well as the only ARG on co-occurring bacteria from the *Aphanizomenon* and *Cuspidothrix* cultures. The *adeF* gene codifies to a membrane fusion protein of the multidrug efflux complex AdeFGH. Bacteria exhibiting this gene have shown enhanced resistance to fluoroquinolone and tetracycline antibiotics, as well as chloramphenicol, clindamycin, trimethoprim, sulfamethoxazole and sodium dodecyl sulfate, and dyes such as ethidium bromide, safranin O and acridine orange [49]. The identification of the *adeF*-type gene in the studied cyanobacterial genomes could thus explain the reduced susceptibility phenotype of freshwater *Chrysosporum bergii* (LMECYA 246) and *Aphanizomenon gracile* (LMECYA 40) to trimethoprim and nalidixic acid, as we previously described [9]. In a previous study, we also reported the reduced susceptibility of *Planktothrix agardhii* and *Planktothrix mougeotti* strains to trimethoprim, norfloxacin and nalidixic acid [10]. However, in the present study, we did not detect the *adeF* gene in these species, which suggests another mechanism underlying this resistant phenotype, including intrinsic resistance, as we previously hypothesized [10]. It is interesting to note that in 9 out of the 11 *adeF*-type genes detected on the cyanobacterial binned genomes, the gene was also present in bins belonging to bacteria on the same sample, suggesting HGT of this ARG under selective pressure. Although no direct genetic evidence of HGT, such as ARGs within mobile genetic elements or flanking insertion sequences, was identified in this study, this co-occurrence provided indirect support for HGT between the cyanobacteria and co-existing bacteria. Future studies using long-read sequencing and functional validation assays are required to confirm the mobility and expression of these ARGs. Nevertheless, the potential for ARG mobility was already predicted in cyanobacterial genomes by Timms et al. [16], namely for genes associated with resistance to aminoglycosides and beta-lactams. Previously, Wang and Hong [50] reported the ubiquity of *adeF* and other efflux pump-associated ARGs in samples from reclaimed water distribution systems, namely from POU (Point of Use). These authors hypothesized that the enrichment of these environments with efflux pump mechanisms may be due to chemical stressors (like chlorine) and/or to antibiotic residues that are commonly present in the water samples [50]. Indeed, quinolones and tetracyclines are relevant prescribed antibiotics in human and veterinary medicine in Portugal, respectively [51], so it is not surprising that they are important freshwater contaminants underlying resistance mechanisms in the environment.

Another potentially interesting gene detected in four *Planktothrix* samples (*P. agardhii* LMECYA 280; *P. mougeotii* LEGE 06224, LEGE 06226 and LEGE 07231) was *ermF*. The *ermF* gene encodes for a protein that confers cross-resistance to macrolides, lincosamides and streptogramin B (MLSb) [52], meaning that it is part of the RNA methyltransferase family of the 23S ribosomal RNA conferring degrees of the MLSb phenotype [53]. Considering the clinical relevance, prevalence in the environment, association with MGE and HGT potential of the *ermF* gene [54], we hypothesize that cyanobacteria might acquire this gene from their bacterial neighbors in aquatic habitats, contributing to their putative role in the aquatic resistome. Indeed, *ermF* was suggested as a genetic indicator for assessing resistance to macrolides in the environment [54]. Actually, Timms et al. [16] also found an *erm* gene (*ermC*) associated with a plasmid replicon in the genome of the cyanobacteria *C. issatschenkoi* CHARLIE-1.

In the present study, we also found that two out of four occurrences of the *ermF*-type gene in our *Planktothrix* samples appeared associated with *mefC*-type and *mphG*-type genes. This association was also present in a bacterium from our *P. mougeotii* LEGE 06225 culture. The *mefC* gene encodes for a macrolide efflux pump capable of facilitating macrolide antibiotics out of the cell, while *mphG* encodes for a macrolide 2′-phosphotransferase responsible for the inactivation of macrolide antibiotics. These genes have previously been described as transferring together [55] as part of a multidrug plasmid from the species *Photobacterium damselae*, a Gram-negative bacteria that is pathogenic for marine organisms. Nonaka et al. [55] tested how the presence of these genes together on *E. coli* cells would affect the minimum inhibitory concentration (MIC) of antibiotics on the samples. They found that *E. coli* started displaying “*high-level macrolide resistance* via *combined expression of the efflux pump and macrolide phosphotransferase*”. The observations made by these authors represent the first registered occurrence of this novel gene pair on marine bacteria [55]. To our knowledge, our results also represent the first report of this gene pair on freshwater cyanobacterial cells. The resistance phenotype of the studied cyanobacteria to macrolides should be further investigated considering that we did not include antibiotics from this class in our previous studies. Our results also hint at a possible new association with *ermF*, reinforcing the resistance to macrolides even further. This association should be deeply studied on *Planktothrix* using targeted sequencing methods in order for it to be better understood.

In our study, it is also interesting to note that macrolide resistance genes were mainly detected in cyanobacteria/bacteria isolated from the WWTP. Indeed, a report of antibiotic residues in the WWTP final effluents of seven European countries showed that the macrolides azithromycin and clarithromycin were ubiquitous, presenting their maximal concentrations in Portuguese samples (1577.3 ng/L and 346.8 ng/L, respectively) [56]. However, in parallel to the WWTP, we cannot exclude the hypothesis that freshwater cyanobacteria (and their associated bacteria) may develop macrolide resistance mechanisms, considering that they could be exposed to macrolide residues originating from veterinary use that could end up in surface freshwater reservoirs located in rural areas [57]. In fact, erythromycin, clarithromycin and azithromycin were included in the 2015 and 2018 EU Watch List of potentially hazardous compounds for the aquatic environment, meaning that their occurrence in the environment creates a potential risk for humans and other living organisms, but the knowledge about this risk is not sufficient. They were removed from the 2020 revised version of this list, but they are still categorized as Highest Priority/Critically Important Antimicrobials by the WHO [57]. In addition, the biotic and abiotic degradation of macrolides in the environment can lead to the formation of by-products with under-investigated ecotoxicological impacts [58].

In summary, our research on ARGs in cyanobacterial genomes revealed the presence of four variants conferring resistance in pathogenic bacteria to tetracyclines, fluoroquinolones (*adeF*-type) and macrolides (*ermF*-type, *mefC*-type and *mphG*-type). The gene a*deF*-type was found across genomes from the Nostocales order. The genes *ermF*-type, *mefC*-type and *mphG*-type were found across Oscillatoriales genomes. Interestingly, Timms et al. [16] recently predicted ARGs conferring resistance to several antibiotics (β-lactams, chloramphenicols, tetracyclines, macrolides and aminoglycosides) in 10% of a total of 862 analyzed cyanobacterial genomes, with Nostocales (23%) and Oscillatoriales (8%) genomes being those with the highest percentage of ARGs.

Our results also revealed that all the other detected ARGs were located in genomic bins belonging to bacteria present in the cyanobacterial cultures under study. The *sul1* gene was the most conserved ARG found in our samples, being detected in four different samples with similar sequence lengths and perfect identity scores related to those of the CARD reference variant. This gene confers resistance to sulphonamides via antibiotic target replacement and has been described as abundant worldwide and present in a large variety of species [59,60]. Another interesting result found in six bacteria from the cyanobacterial cultures was the *aph*(*3*″)-*Ib*-type, *aph*(*6*)-*Id*-type and *floR*-type association. APH(3″)-Ib and APH(6)-Id promote the inactivation of aminoglycoside antibiotics, and their simultaneous presence has been strongly associated with streptomycin resistant phenotypes in *Corynebacterium striatum* [61], an emerging multidrug-resistant skin and mucous membrane pathogen [62]. The *floR* gene, on the other hand, is a well-described efflux pump gene conferring resistance to phenicol [63]. The reason for this association between the three genes is uncertain. One of the plausible explanations is that it presents a fitness advantage in a wastewater environment with the presence of multiple antibiotics, and were then transferred through a multidrug resistance plasmid. All these ARGs, while not found in cyanobacterial genomes, reflect the potential for gene exchange in shared environments and reinforce the ecological relevance of studying cyanobacteria within their microbial associations.

Together with other studies [12,13,14,15,16], our results put in evidence the role of cyanobacteria and their associated bacterial communities as potential drivers of antibiotic resistance in water environments. Furthermore, the other partial ARG hits detected on the cyanobacterial bins can also be used as a guide in choosing antibiotics to be screened in phenotypic approaches with cyanobacteria strains. These partial hits can also eventually be used in new studies for the discovery of putative new ARGs.

This newly developed CyanoPipeline integrates quality filtering, assembly, taxonomic classification, genome binning and ARG prediction into a unified and fully automated workflow. This comprehensive approach enhances reproducibility and significantly reduces manual intervention compared to conventional multi-tool pipelines. Unlike general-purpose metagenomic workflows such as Anvi’o [64], MetaWRAP [65] and ATLAS [66], which require extensive user input and lack optimizations for cyanobacterial genomes, CyanoPipeline incorporates a custom bin selection algorithm based on weighted GC content and genome size, improving its accuracy in identifying target cyanobacterial bins and minimizing user bias. Utilizing MaxBin2 to bin contigs as short as 500 bp enables the effective recovery of fragmented genomes from complex, non-axenic cultures. The pipeline successfully recovered 41 cyanobacterial genomes, 26 of which met high-quality standards (completeness ≥ 90%, contamination ≤ 5%), achieving recovery rates that match or exceed those reported in comparable metagenomic studies [16,65,67,68]. Moreover, by integrating ARG prediction alongside genome reconstruction, the pipeline offers a scalable and reproducible solution tailored for high-throughput environmental resistome analyses in cyanobacterial metagenomes.

A final note to mention is that as a parallel output, this work is also useful for the reclassification of some cyanobacteria strains from the ESSACC culture collection, as referred to in the Section 3.1. The misidentification of cyanobacteria is a common situation, being particularly problematic in comparative studies. In the case of AMR, it may lead to the biased interpretation of the ARG distribution among different genus/species, which may compromise the identification of species- or genus-specific resistance traits, such as intrinsic antibiotic resistance or the potential for ARG transfer.

## 5. Conclusions

In this study, we use a new genome binning approach to identify ARGs in environmental (freshwater and wastewater) cyanobacteria. The whole genome sequencing of non-axenic cultures of cyanobacteria represents a straightforward, accurate and effective strategy to explore genomic features in hard-to-isolate organisms, given the availability of good sequencing depth assemblies. This procedure enabled us to overcome the complexity and specificity of non-axenic cyanobacteria cultures and allowed us to obtain 26 high-quality cyanobacterial genomes out of 41 sequenced cultures. Our results also indicate that cyanobacteria might play a putative role in the emergence and/or dissemination of ARGs since, though presenting a low diversity of ARG types, several variants of known ARGs were detected across the samples from distinct genera of cyanobacteria: *adeF*-type, *ermF*-type, *mphG*-type and *mefC*-type; these genes had never been previously described on freshwater cyanobacterial genomes, which is a significant contribution to the present field of the aquatic (cyano)resistome.

Overall, these findings highlight the importance of studying the role of cyanobacteria in the environmental resistome from a One Health perspective. Indeed, increasing evidence points out the participation of cyanobacteria and their associated phycosphere in the cycle of AR dissemination in aquatic environments. Cyanobacteria blooms may function both as receptors of ARGs (e.g., from livestock pathogenic bacteria in grazing areas or from wastewater discharges) and as vehicles for ARG dissemination to animals (livestock, as well as wild and companion animals) and humans through exposure in surface freshwater reservoirs. On the other hand, a possible role of cyanobacterial toxins in exacerbating HGT in ARGs in microbial biofilms in drinking water treatment plants and the potential stimulation of cyanotoxin production by antibiotics have also been considered as additional issues when considering the complexity of the relation between cyanobacteria and antibiotic pollution (antibiotic residues, resistant bacteria, eDNA) [69]. In this context, cyanobacterial blooms should be considered in the scope of AMR monitoring in water bodies, namely in surface freshwaters where antibiotic pollution is a concern.

## Figures and Tables

**Table 1:** Strict (blue box) and Perfect (yellow box) hits for antibiotic resistance genes, as well as their respective resistance mechanisms, using the RGI tool on cyanobacterial and bacterial genome bins, from non-axenic cyanobacteria cultures. Each row of this Table corresponds to cyanobacterial samples, while each column corresponds to the genomic bins generated by MaxBin2 for each sample. *ermF*/*mefC*/*mphG* and *aph*(*3"*)-*Ib*/*aph*(*6*)-*Id*/*floR* associations are marked with a blue square. *—*aadA6*/*aadA10*; CB—genes detected on cyanobacterial genome; B—genes detected on bacterial genomes; ND—not detected.

Strain	Antibiotic Efflux										Antibiotic Inactivation																		**Antibiotic Target Alteration/Replacement**			
	*adeF*		*mefC*		*tet D*		*qacH*		*floR*		*mphG*		*aadA2*		*aadA8*		*aadA13*		*aadA16*		*aadA6/A10* *		*aph(3″)-Ib*		*aph(6)-Id*		*ampS*		***ermF***		***sul1***	
	CB	B	CB	B	CB	B	CB	B	CB	B	CB	B	CB	B	CB	B	CB	B	CB	B	CB	B	CB	B	CB	B	CB	B	CB	B	CB	B
**LMECYA 161**	ND	3	ND	1	ND	ND	ND	1	ND	ND	ND	1	ND	ND	ND	ND	ND	ND	ND	1	ND	ND	ND	ND	ND	ND	ND	ND	ND	1	ND	1
**LMECYA 165**	ND	1	ND	ND	ND	ND	ND	ND	ND	ND	ND	ND	ND	ND	ND	ND	ND	ND	ND	ND	ND	ND	ND	ND	ND	ND	ND	ND	ND	ND	ND	ND
**LMECYA 182**	1	ND	ND	ND	ND	ND	ND	ND	ND	ND	ND	ND	ND	ND	ND	ND	ND	ND	ND	ND	ND	ND	ND	ND	ND	ND	ND	ND	ND	ND	ND	ND
**LMECYA 213**	ND	2	ND	ND	ND	ND	ND	ND	ND	ND	ND	ND	ND	ND	ND	ND	ND	ND	ND	ND	ND	ND	ND	ND	ND	ND	ND	ND	ND	ND	ND	ND
**LMECYA 246**	1	3	ND	ND	ND	1	ND	ND	ND	ND	ND	ND	ND	ND	ND	ND	ND	ND	ND	ND	ND	ND	ND	ND	ND	ND	ND	1	ND	ND	ND	ND
**LMECYA 313**	1	1	ND	ND	ND	ND	ND	ND	ND	ND	ND	ND	ND	ND	ND	ND	ND	ND	ND	ND	ND	ND	ND	ND	ND	ND	ND	ND	ND	ND	ND	ND
**LMECYA 009**	1	1	ND	ND	ND	ND	ND	ND	ND	ND	ND	ND	ND	ND	ND	ND	ND	ND	ND	ND	ND	ND	ND	ND	ND	ND	ND	ND	ND	ND	ND	ND
**LMECYA 031**	1	2	ND	ND	ND	ND	ND	ND	ND	ND	ND	ND	ND	ND	ND	ND	ND	ND	ND	ND	ND	ND	ND	ND	ND	ND	ND	ND	ND	ND	ND	ND
**LMECYA 040**	1	ND	ND	ND	ND	ND	ND	ND	ND	ND	ND	ND	ND	ND	ND	ND	ND	ND	ND	ND	ND	ND	ND	ND	ND	ND	ND	ND	ND	ND	ND	ND
**LMECYA 089**	1	1	ND	ND	ND	ND	ND	ND	ND	ND	ND	ND	ND	ND	ND	ND	ND	ND	ND	ND	ND	ND	ND	ND	ND	ND	ND	ND	ND	ND	ND	ND
**LMECYA 190**	1	4	ND	ND	ND	ND	ND	ND	ND	ND	ND	ND	ND	ND	ND	ND	ND	ND	ND	ND	ND	ND	ND	ND	ND	ND	ND	ND	ND	ND	ND	ND
**LMECYA 237**	1	4	ND	ND	ND	ND	ND	ND	ND	ND	ND	ND	ND	ND	ND	ND	ND	ND	ND	ND	ND	ND	ND	ND	ND	ND	ND	ND	ND	ND	ND	ND
**LMECYA 253**	1	1	ND	ND	ND	ND	ND	ND	ND	ND	ND	ND	ND	ND	ND	ND	ND	ND	ND	ND	ND	ND	ND	ND	ND	ND	ND	ND	ND	ND	ND	ND
**LMECYA 328**	1	1	ND	ND	ND	ND	ND	ND	ND	ND	ND	ND	ND	ND	ND	ND	ND	ND	ND	ND	ND	ND	ND	ND	ND	ND	ND	ND	ND	ND	ND	ND
**LMECYA 153A**	ND	2	ND	ND	ND	ND	ND	1	ND	ND	ND	ND	ND	ND	ND	ND	ND	ND	ND	ND	ND	1	ND	ND	ND	ND	ND	ND	ND	ND	ND	ND
**LMECYA 230**	ND	2	ND	ND	ND	ND	ND	1	ND	2	ND	ND	ND	ND	ND	1	ND	1	ND	ND	ND	ND	ND	2	ND	1	ND	ND	ND	ND	ND	1
**LMECYA 257**	ND	1	ND	ND	ND	ND	ND	ND	ND	ND	ND	ND	ND	ND	ND	ND	ND	ND	ND	ND	ND	ND	ND	ND	ND	ND	ND	ND	ND	ND	ND	ND
**LMECYA 280**	ND	2	ND	ND	ND	ND	ND	1	ND	ND	ND	ND	ND	ND	ND	ND	ND	1	ND	ND	ND	ND	ND	ND	ND	ND	ND	ND	1	ND	ND	1
**LMECYA 283**	ND	4	ND	1	ND	ND	ND	ND	ND	1	ND	1	ND	ND	ND	ND	ND	ND	ND	ND	ND	ND	ND	1	ND	1	ND	ND	ND	ND	ND	ND
**LMECYA 292**	ND	ND	ND	ND	ND	ND	ND	ND	ND	ND	ND	ND	ND	ND	ND	ND	ND	ND	ND	ND	ND	ND	ND	1	ND	1	ND	ND	ND	ND	ND	ND
**LEGE 06224**	ND	4	1	ND	ND	ND	ND	1	ND	1	1	ND	ND	ND	ND	ND	ND	ND	ND	1	ND	ND	ND	1	ND	1	ND	ND	1	ND	ND	1
**LEGE 06225**	ND	2	ND	1	ND	ND	ND	ND	ND	ND	ND	1	ND	ND	ND	ND	ND	ND	ND	ND	ND	ND	ND	1	ND	1	ND	ND	ND	1	ND	ND
**LEGE 06226**	ND	3	1	ND	ND	ND	ND	ND	ND	1	1	ND	ND	ND	ND	ND	ND	ND	ND	ND	ND	ND	ND	1	ND	1	ND	1	1	ND	ND	ND
**LEGE 06233**	ND	1	ND	1	ND	ND	ND	ND	ND	1	ND	ND	ND	ND	ND	ND	ND	ND	ND	ND	ND	ND	ND	1	ND	1	ND	ND	ND	ND	ND	ND
**LEGE 07227**	ND	2	ND	ND	ND	ND	ND	1	ND	1	ND	ND	ND	ND	ND	ND	ND	ND	ND	1	ND	ND	ND	1	ND	1	ND	ND	ND	1	ND	1
**LEGE 07229**	ND	1	ND	ND	ND	ND	ND	1	ND	ND	ND	ND	ND	1	ND	ND	ND	ND	ND	ND	ND	ND	ND	ND	ND	ND	ND	ND	ND	ND	ND	ND
**LEGE 07231**	ND	ND	ND	ND	ND	ND	ND	ND	ND	ND	ND	1	ND	ND	ND	ND	ND	ND	ND	ND	ND	ND	ND	1	ND	ND	ND	ND	1	ND	ND	ND

## Data Availability

The raw sequence reads used for the assembly were deposited in the National Center for Biotechnology Information (NCBI) Sequence Read Archive under accession numbers SAMN34233939 to SAMN34233979 (Bioproject ref. PRJNA956929).

## Supplementary Materials

The following supporting information can be downloaded at https://www.mdpi.com/article/10.3390/microorganisms13061252/s1, Figure S1: Map of cyanobacteria sampling sites (see Supplementary Table S1 for strain identification). Sampling site 21 corresponds to a wastewater treatment plant. All the other sites correspond to surface freshwaters from rivers (8 and 19) and reservoirs (all remaining). Figure S2: Sankey diagrams of the taxa identified with Kraken2 for cyanobacterial metagenomic samples. Table S1: Cyanobacteria strains’ taxonomy according to (a) microscopic identification (morphology); (b) genomic classification using Kraken2 software, and the accession number of each strain is also indicated (NCBI BioProject ref. PRJNA956929); (c) reclassification based on morphologic and genomic analysis, and taxonomic revisions. Table S2: Percentage of classified cyanobacterial metagenome reads that present the best alignment with each genus. Based on the reads that match the reference 16S sequences belonging to said genus. Intensity of cell coloring is related to the proportion of reads on a sample that are attributed to a given genus. Higher color intensity denotes highest correspondences. Table S3: CheckM reports for the 41 analyzed recovered cyanobacteria genomes. Table S4: Per-base genome coverage of recovered cyanobacteria genomes as the average number of reads displaying information about each nucleotide.

## References

[1] Banin E., Hughes D., Kuipers O.P. (2017). Bacterial pathogens, antibiotics and antibiotic resistance. FEMS Microbiol. Rev..

[2] (2021). WHO Antibiotic Resistance. https://www.who.int/news-room/fact-sheets/detail/antibiotic-resistance.

[3] Brown E.D., Wright G.D. (2016). Antibacterial drug discovery in the resistance era. Nature.

[4] Surette M.D., Wright G.D. (2017). Lessons from the environmental antibiotic resistome. Annu. Rev. Microbiol..

[5] Wright G.D. (2007). The antibiotic resistome: The nexus of chemical and genetic diversity. Nat. Rev. Microbiol..

[6] Baquero F., Martínez J.-L., Cantón R. (2008). Antibiotics and antibiotic resistance in water environments. Curr. Opin. Biotechnol..

[7] Zhang X.-X., Zhang T., Fang H.H. (2009). Antibiotic resistance genes in water environment. Appl. Microbiol. Biotechnol..

[8] Caniça M., Manageiro V., Jones-Dias D., Clemente L., Gomes-Neves E., Poeta P., Dias E., Ferreira E. (2015). Current perspectives on the dynamics of antibiotic resistance in different reservoirs. Res. J. Microbiol..

[9] Dias E., Oliveira M., Jones-Dias D., Vasconcelos V., Ferreira E., Manageiro V., Caniça M. (2015). Assessing the antibiotic susceptibility of freshwater Cyanobacteria spp.. Front. Microbiol..

[10] Dias E., Oliveira M., Manageiro V., Vasconcelos V., Caniça M. (2019). Deciphering the role of cyanobacteria in water resistome: Hypothesis justifying the antibiotic resistance (phenotype and genotype) in *Planktothrix* genus. Sci. Total Environ..

[11] Cornet L., Bertrand A.R., Hanikenne M., Javaux E.J., Wilmotte A., Baurain D. (2018). Metagenomic assembly of new (sub)polar Cyanobacteria and their associated microbiome from non-axenic cultures. Microb. Genom..

[12] Guo Y., Liu M., Liu L., Liu X., Chen H., Yang J. (2018). The antibiotic resistome of free-living and particle-attached bacteria under a reservoir cyanobacterial bloom. Environ. Int..

[13] Zhang Q., Zhang Z., Lu T., Peijnenburg W.J.G.M., Gillings M., Yang X., Chen J., Penuelas J., Zhu Y.-G., Zhou N.-Y. (2020). Cyanobacterial blooms contribute to the diversity of antibiotic-resistance genes in aquatic ecosystems. Commun. Biol..

[14] Wang Z., Chen Q., Zhang J., Guan T., Chen Y., Shi W. (2020). Critical roles of cyanobacteria as reservoir and source for antibiotic resistance genes. Environ. Int..

[15] Wang Z., Chen Q., Zhang J., Yan H., Chen Y., Chen C., Chen X. (2021). High prevalence of unstable antibiotic heteroresistance in cyanobacteria causes resistance underestimation. Water Res..

[16] Timms V.J., Hassan K.A., Pearson L.A., Neilan B.A. (2023). Cyanobacteria as a critical reservoir of the environmental antimicrobial resistome. Environ. Microbiol..

[17] Rocha E.P. (2006). Inference and analysis of the relative stability of bacterial chromosomes. Mol. Biol. Evol..

[18] Zhaxybayeva O., Gogarten J.P., Charlebois R.L., Doolittle W.F., Papke R.T. (2006). Phylogenetic analyses of cyanobacterial genomes: Quantification of horizontal gene transfer events. Genome Res..

[19] Hernando-Amado S., Coque T.M., Baquero F., Martínez J.L. (2019). Defining and combating antibiotic resistance from One Health and Global Health perspectives. Nat. Microbiol..

[20] Paulino S., Sam-Bento F., Churro C., Alverca E., Dias E., Valério E., Pereira P. (2009). The Estela Sousa e Silva Algal Culture Collection: A resource of biological and toxicological interest. Hydrobiologia.

[21] Martins J., Peixe L., Vasconcelos V. (2010). Cyanobacteria and bacteria co-occurrence in a wastewater treatment plant: Absence of allelopathic effects. Water Sci. Technol..

[22] Ramos V., Morais J., Castelo-Branco R., Pinheiro Â., Martins J., Regueiras A., Pereira A.L., Lopes V.R., Frazão B., Gomes D. (2018). Cyanobacterial diversity held in microbial biological resource centers as a biotechnological asset: The case study of the newly established LEGE culture collection. J. Appl. Phycol..

[23] Skulberg R., Skulberg O.M. (1990). Forskning MedAlgekulturerNIVAs Kultursampling av Alger.

[24] (2006). Water Quality—Guidance Standard on the Enumeration of Phytoplankton Using Inverted Microscopy (Utermöhl Technique).

[25] Wood D.E., Lu J., Langmead B. (2019). Improved metagenomic analysis with Kraken 2. Genome Biol..

[26] Quast C., Pruesse E., Yilmaz P., Gerken J., Schweer T., Yarza P., Peplies J., Glöckner F.O. (2013). The SILVA ribosomal RNA gene database project: Improved data processing and web-based tools. Nucleic Acids Res..

[27] Bankevich A., Nurk S., Antipov D., Gurevich A.A., Dvorkin M., Kulikov A.S., Lesin V.M., Nikolenko S.I., Pham S., Prjibelski A.D. (2012). SPAdes: A new genome assembly algorithm and its applications to single-cell sequencing. J. Comput. Biol..

[28] Wu Y.-W., Simmons B.A., Singer S.W. (2016). MaxBin 2.0: An automated binning algorithm to recover genomes from multiple metagenomic datasets. Bioinformatics.

[29] Altschul S.F., Gish W., Miller W., Myers E.W., Lipman D.J. (1990). Basic local alignment search tool. J. Mol. Biol..

[30] Gurevich A., Saveliev V., Vyahhi N., Tesler G. (2013). QUAST: Quality assessment tool for genome assemblies. Bioinformatics.

[31] Li H. (2013). Aligning sequence reads, clone sequences and assembly contigs with BWA-MEM. arXiv.

[32] Li H., Handsaker B., Wysoker A., Fennell T., Ruan J., Homer N. (2009). The sequence alignment/map format and SAMtools. Bioinformatics.

[33] Milne I., Stephen G., Bayer M., Cock P.J., Pritchard L., Cardle L., Shaw P.D., Marshall D. (2013). Using Tablet for visual exploration of second-generation sequencing data. Brief Bioinform.

[34] Parks D.H., Imelfort M., Skennerton C.T., Hugenholtz P., Tyson G.W. (2015). CheckM: Assessing the quality of microbial genomes recovered from isolates, single cells, and metagenomes. Genome Res..

[35] Jia B., Raphenya A.R., Alcock B., Waglechner N., Guo P., Tsang K.K., Lago B.A., Dave B.M., Pereira S., Sharma A.N. (2016). CARD 2017: Expansion and model-centric curation of the comprehensive antibiotic resistance database. Nucleic Acids Res..

[36] Alcock B.P., Raphenya A.R., Lau T.T.Y., Tsang K.K., Bouchard M., Edalatmand A., Huynh W., Nguyen A.-L.V., Cheng A.A., Liu S. (2020). CARD 2020: Antibiotic resistome surveillance with the comprehensive antibiotic resistance database. Nucleic Acids Res..

[37] Hrycik A.R., Shambaugh A., Stockwell J.D. (2019). Comparison of FlowCAM and microscope biovolume measurements for a diverse freshwater phytoplankton community. J. Plankton Res..

[38] Figueiredo D. (2025). The Need to Increase Strain-Specific DNA Information from the Invasive Cyanobacteria *Sphaerospermopsis aphanizomenoides* and *Cuspidothrix issatschenkoi*. Water.

[39] Wacklin P., Hoffmann L., Komárek J. (2009). Nomenclatural validation of the genetically revised cyanobacterial genus *Dolichospermum* (Ralfs ex Bornet et Flahault) comb. Nova. Fottea.

[40] Li X., Dreher T.W., Li R. (2016). An overview of diversity, occurrence, genetics and toxin production of bloom-forming *Dolichospermum* (*Anabaena*) species. Harmful Algae.

[41] Rajaniemi P., Komárek J., Willame R., Hrouzek P., Kaštovská K., Hoffmann L., Sivonen K. (2005). Taxonomic consequences from the combined molecular and phenotype evaluation of selected *Anabaena* and *Aphanizomenon* strains. Agol. Stud. Arch. Für Hydrobiol..

[42] Toporowska M., Pawlik-Skowrońska B., Kalinowska R. (2016). Mass Development of Diazotrophic Cyanobacteria (Nostocales) and Production of Neurotoxic Anatoxin-a in a *Planktothrix* (Oscillatoriales) Dominated Temperate Lake. Water Air Soil Pollut.

[43] Cao H., Shimura Y., Masanobu K., Yin Y. (2014). Draft Genome Sequence of the Toxic Bloom-Forming Cyanobacterium *Aphanizomenon flos-aquae* NIES-81. Genome Announc..

[44] Wang H., Sivonen K., Rouhiainen L., Fewer D.P., Lyra C., Rantala-Ylinen A., Vestola J., Jokela J., Rantasärkkä K., Li Z. (2012). Genome-derived insights into the biology of the hepatotoxic bloom-forming cyanobacterium *Anabaena* sp. strain 90. BMC Genom..

[45] Suzuki S., Yamaguchi H., Kawachi M. (2019). Draft Genome Sequences of Three Filamentous Cyanobacterial Strains, *Dolichospermum planctonicum* NIES-80, *Planktothrix agardhii* NIES-905, and *Sphaerospermopsis reniformis* NIES-1949. Microbiol. Resour. Announc..

[46] Tooming-Klunderud A., Sogge H., Rounge T.B., Nederbragt A.J., Lagesen K., Glöckner G., Hayes P.K., Rohrlack T., Jakobsen K.S. (2013). From green to red: Horizontal gene transfer of the phycoerythrin gene cluster between *Planktothrix* strains. Appl. Environ. Microbiol..

[47] Tanabe Y., Yamaguchi H. (2019). Draft genome sequence of *Microcystis aeruginosa* NIES-, 4285, isolated from brackish water (Lake Abashiri, Japan). Microbiol. Resour. Announc..

[48] Parks D.H., Rinke C., Chuvochina M., Chaumeil P.-A., Woodcroft B.J., Evans P.N., Hugenholtz P., Tyson G.W. (2017). Recovery of nearly 8,000 metagenome-assembled genomes substantially expands the tree of life. Nat. Microbiol..

[49] Coyne S., Rosenfeld N., Lambert T., Courvalin P., Périchon B. (2010). Overexpression of resistance-nodulation-cell division pump AdeFGH confers multidrug resistance in *Acinetobacter baumannii*. Antimicrob. Agents Chemother..

[50] Wang C., Hong P.-Y. (2020). Genome-resolved metagenomics and antibiotic resistance genes analysis in reclaimed water distribution systems. Water.

[51] Almeida A., Duarte S., Nunes R., Rocha H., Pena A., Meisel L. (2014). Human and veterinary antibiotics used in Portugal—A ranking for ecosurveillance. Toxics.

[52] Kangaba A.A., Saglam F.Y., Tokman H.B., Torun M., Torun M.M. (2015). The prevalence of enterotoxin and antibiotic resistance genes in clinical and intestinal *Bacteroides fragilis* group isolates in Turkey. Anaerobe.

[53] Roberts M.C., Sutcliffe J., Courvalin P., Jensen L.B., Rood J., Seppala H. (1999). Nomenclature for macrolide and macrolide-lincosamide-streptogramin B resistance determinants. Antimicrob. Agents Chemother..

[54] Berendonk T.U., Manaia C.M., Merlin C., Fatta-Kassinos D., Cytryn E., Walsh F., Buergmann H., Sørum H., Norström M., Pons M.-N. (2015). Tackling antibiotic resistance: The environmental framework. Nat. Rev. Microbiol..

[55] Nonaka L., Maruyama F., Suzuki S., Masuda M. (2015). Novel macrolide-resistance genes, mef (C) and mph (G), carried by plasmids from *Vibrio* and *Photobacterium* isolated from sediment and seawater of a coastal aquaculture site. Lett. Appl. Microbiol..

[56] Rodriguez-Mozaz S., Vaz-Moreira I., Della Giustina S.V., Llorca M., Barceló D., Schubert S., Berendonk T.U., Michael-Kordatou I., Fatta-Kassinos D., Martinez J.L. (2020). Antibiotic residues in final effluents of European wastewater treatment plants and their impact on the aquatic environment. Environ. Int..

[57] Trott D.J., Turnidge J., Kovac J.H., Simjee S., Wilson D., Watts J. (2021). Comparative macrolide use in humans and animals: Should macrolides be moved off the World Health Organisation’s critically important antimicrobial list?. J. Antimicrob. Chemother..

[58] Senta I., Kostanjevecki P., Krizman-Matasic I., Terzic S., Ahel M. (2019). Occurrence and behavior of macrolide antibiotics in municipal wastewater treatment: Possible importance of metabolites, synthesis byproducts, and transformation products. J. Environ. Sci. Technol..

[59] Sköld O. (2001). Resistance to trimethoprim and sulfonamides. Vet. Res..

[60] Bandh S.A. (2019). Freshwater Microbiology: Perspectives of Bacterial Dynamics in Lake Ecosystems.

[61] Navas J., Fernández-Martínez M., Salas C., Cano M.E., Martínez-Martínez L. (2016). Susceptibility to Aminoglycosides and Distribution of *aph* and *aac* (3)-XI Genes among *Corynebacterium striatum* Clinical Isolates. PLoS ONE.

[62] Hahn W.O., Werth B.J., Butler-Wu S.M., Rakita R.M. (2016). Multidrug-resistant *Corynebacterium striatum* associated with increased use of parenteral antimicrobial drugs. Emerg. Infect. Dis..

[63] Arcangioli M.-A., Leroy-Sétrin S., Martel J.-L., Chaslus-Dancla E. (1999). A new chloramphenicol and florfenicol resistance gene flanked by two integron structures in *Salmonella typhimurium* DT104. FEMS Microbiol. Lett..

[64] Eren A.M., Esen Ö.C., Quince C., Vineis J.H., Morrison H.G., Sogin M.L., Delmont T.O. (2015). Anvi’o: An advanced analysis and visualization platform for ‘omics data. PeerJ.

[65] Uritskiy G.V., DiRuggiero J., Taylor J. (2018). MetaWRAP—A flexible pipeline for genome-resolved metagenomic data analysis. Microbiome.

[66] Kieser S., Brown J., Zdobnov E.M., Trajkovski M., McCue L.A. (2020). ATLAS: A Snakemake workflow for assembly, annotation, and genomic binning of metagenome sequence data. BMC Bioinform..

[67] Delmont T.O., Eren A.M. (2018). Linking pangenomes and metagenomes: The Prochlorococcus metapangenome. PeerJ.

[68] Anantharaman K., Brown C.T., Hug L.A., Sharon I., Castelle C.J., Probst A.J., Thomas B.C., Burstein D., Thomas B.C., Banfield J.F. (2016). Thousands of microbial genomes shed light on interconnected biogeochemical processes in an aquifer system. Nat. Commun..

[69] Manganelli M., Testai E., Codd G.A. (2025). The complex relationship between cyanobacteria and antibiotics/antimicrobial resistance in the environment: An emerging factor in the One Health vision on antimicrobial resistance. Adv. Oceanogr. Limnol..

