# Supplementary material for: Prediction of Antibiotic Resistance Genes in Cyanobacterial Strains by Whole Genome Sequencing
Source: Microorganisms. 2025 May 28;13(6):1252. doi: 10.3390/microorganisms13061252 (PMC12195311; doi:10.3390/microorganisms13061252)
Supplement: Supplementary file 1 [file microorganisms-13-01252-s001.zip › MS 2828637 Supplementary Figure 1.pdf]

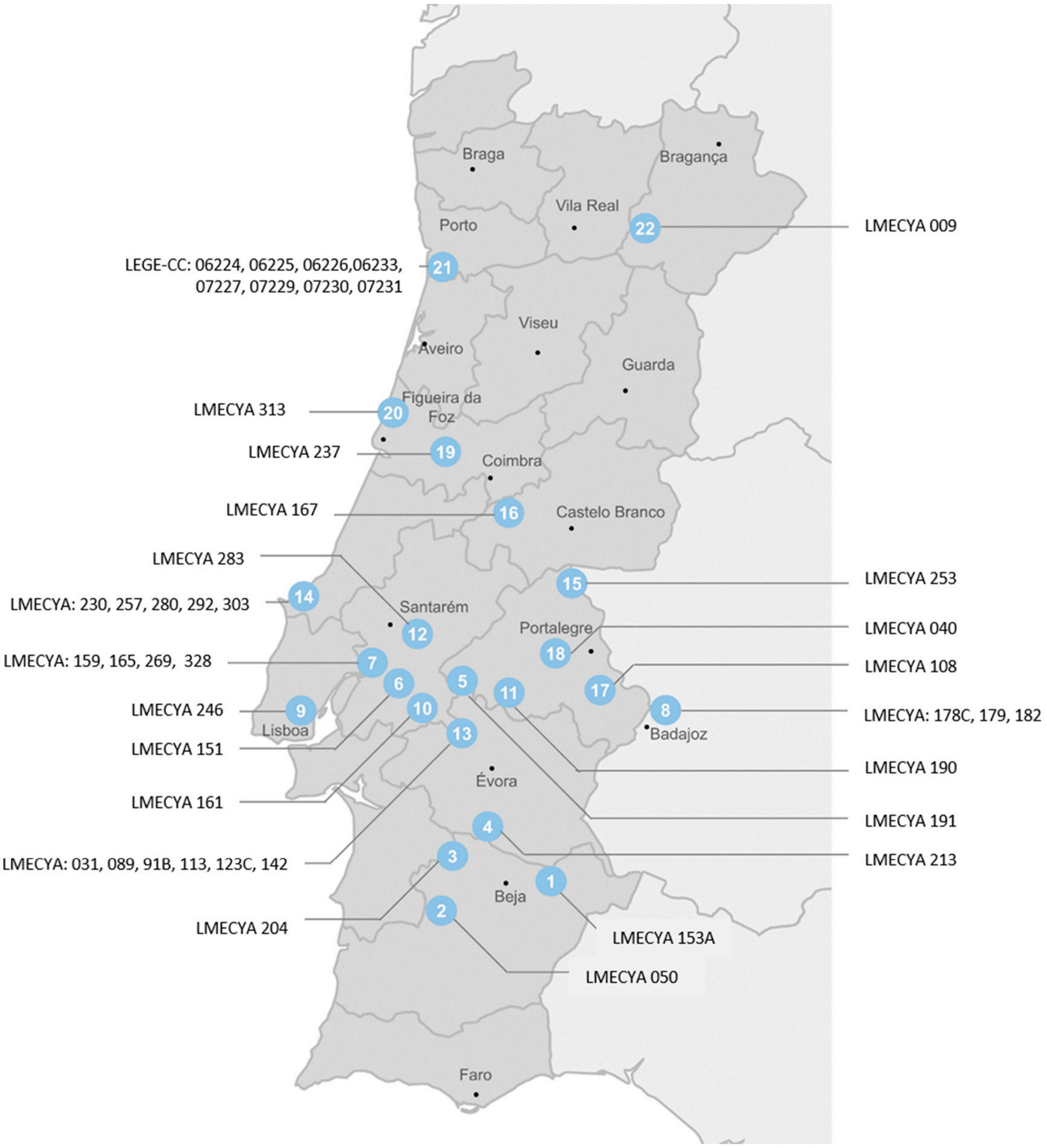

Supplementary Figure 1. Map of cyanobacteria sampling sites (see Supplementary Table 1 to strains identification). Sampling site 21 correspond to a wastewater treatment plant. All the other sites correspond to surface freshwaters from rivers (8 and 19) and reservoirs (all the remaining).
