# Supplementary material for: Prediction of Antibiotic Resistance Genes in Cyanobacterial Strains by Whole Genome Sequencing
Source: Microorganisms. 2025 May 28;13(6):1252. doi: 10.3390/microorganisms13061252 (PMC12195311; doi:10.3390/microorganisms13061252)
Supplement: Supplementary file 1 [file microorganisms-13-01252-s001.zip › MS 2828637 Supplementary Figure 2.pdf]

Supplementary Figure 2. Sankey diagrams of the taxa identified with Kraken2 for cyanobacterial metagenomic samples.

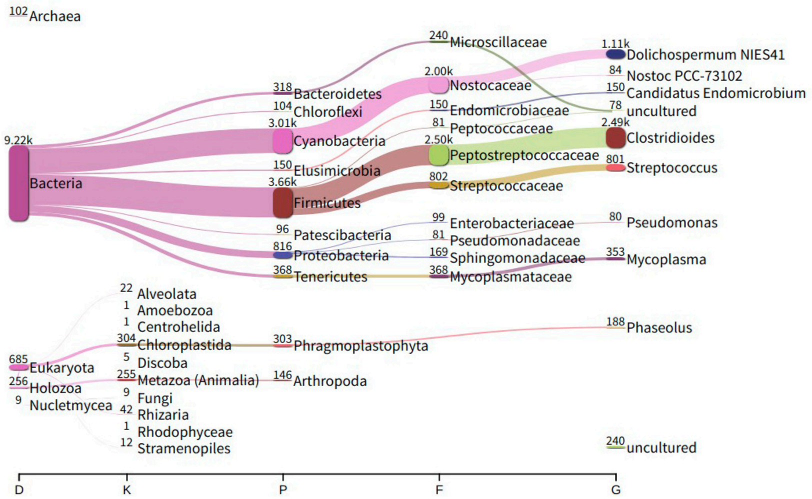

Figure S2.1: Cyanobacterial sample LMECYA 123C

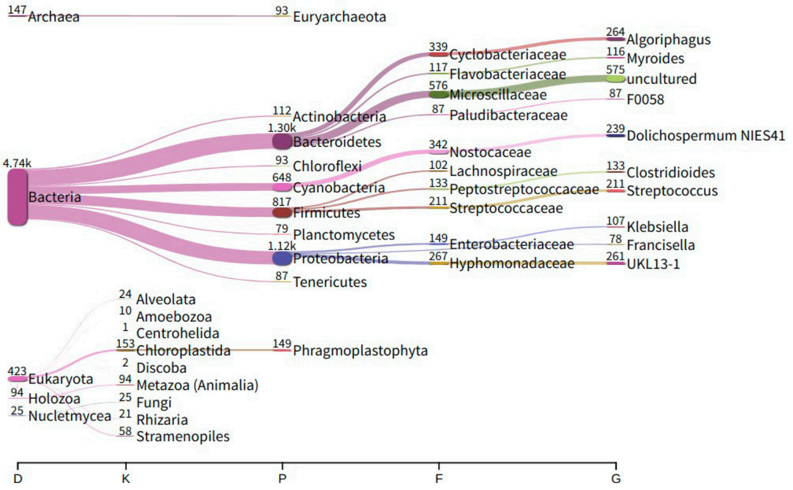

Figure S2.2: Cyanobacterial sample LMECYA 161

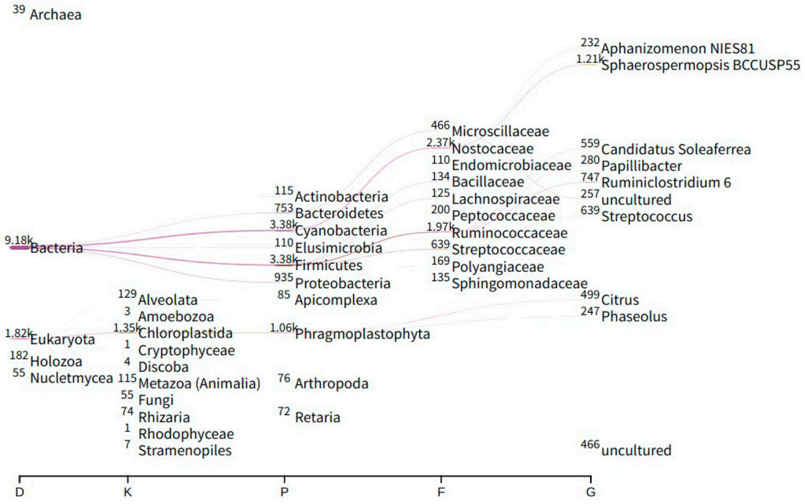

Figure S2.3: Cyanobacterial sample LMECYA 165

Supplementary Figure 2 (cont.)

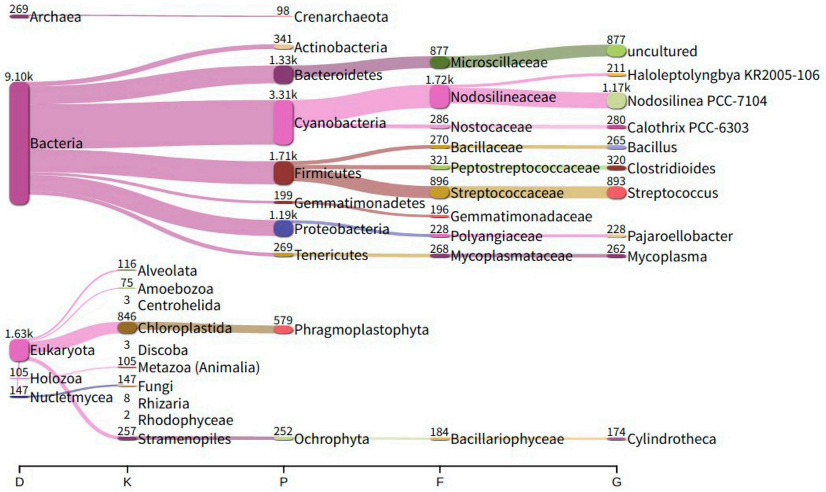

Figure S2.4: Cyanobacterial sample LMECYA 178C

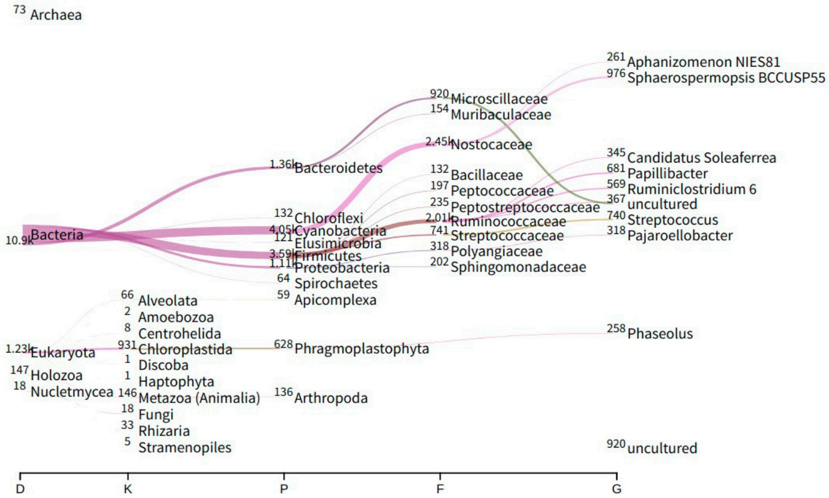

Figure S2.5: Cyanobacterial sample LMECYA 182

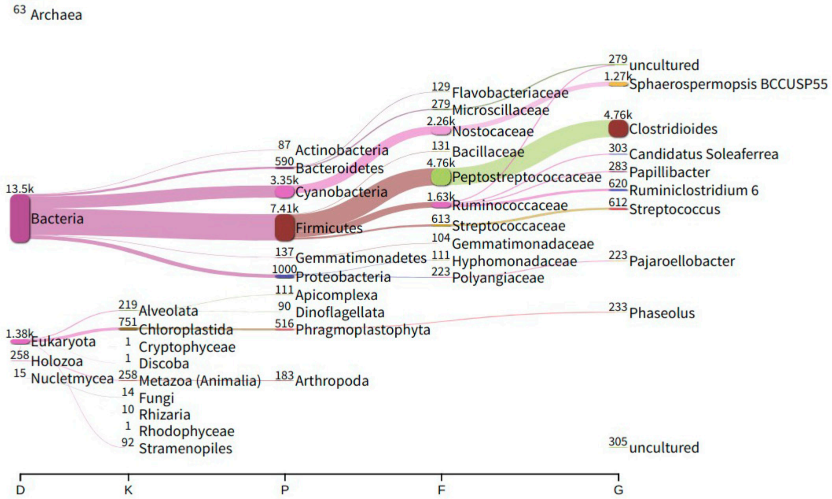

Figure S2.6: Cyanobacterial sample LMECYA 204

Supplementary Figure 2 (cont.)

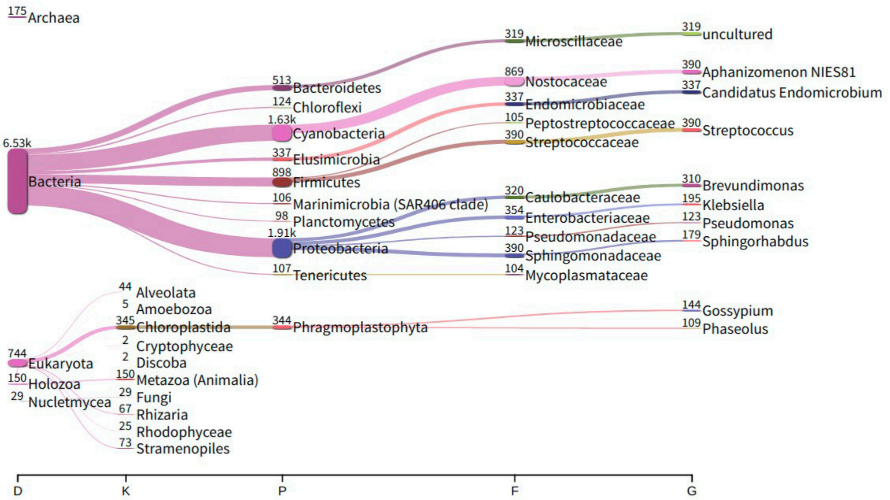

Figure S2.7: Cyanobacterial sample LMECYA 213

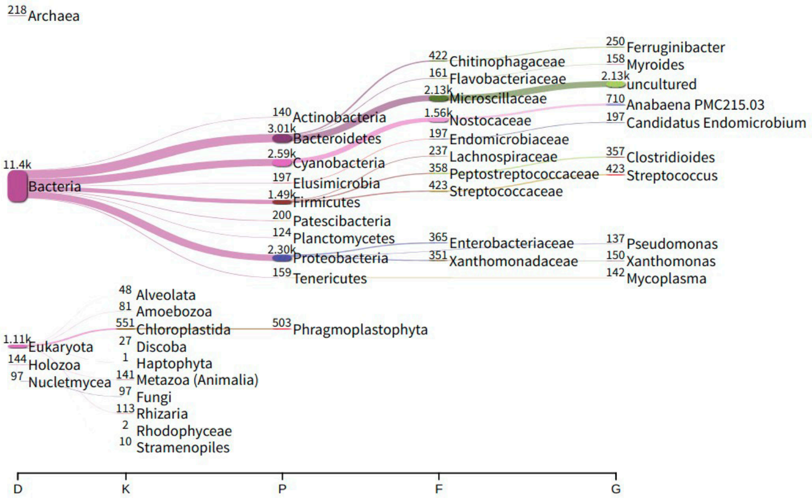

Figure S2.8: Cyanobacterial sample LMECYA 246

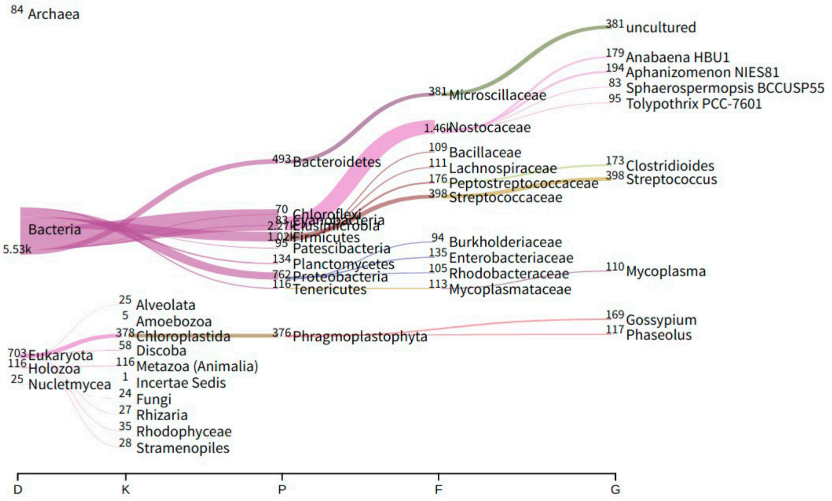

Figure S2.9: Cyanobacterial sample LMECYA 313

Supplementary Figure 2 (cont.)

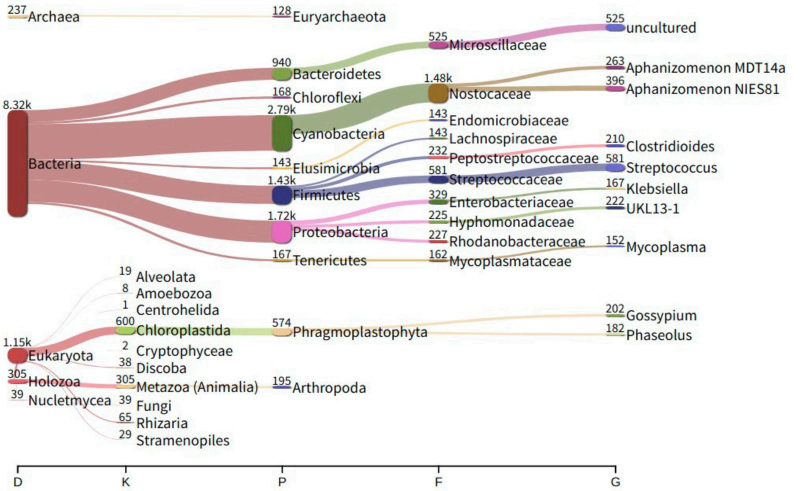

Figure S2.10: Cyanobacterial sample LMECYA 009

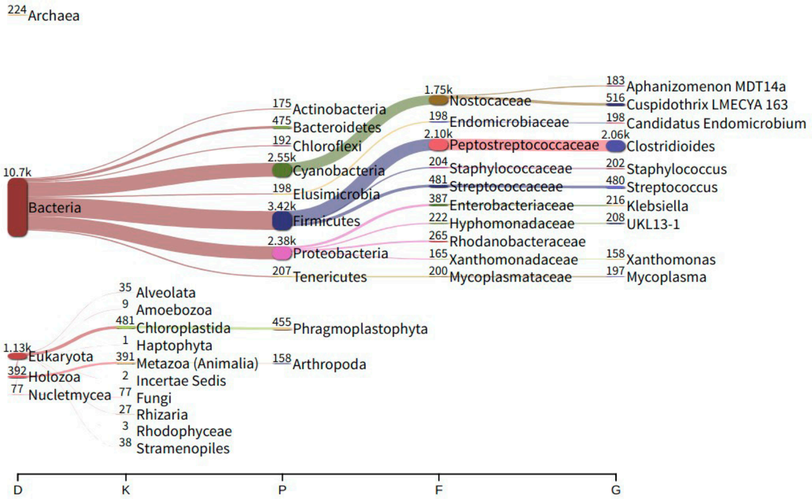

Figure S2.11: Cyanobacterial sample LMECYA 031

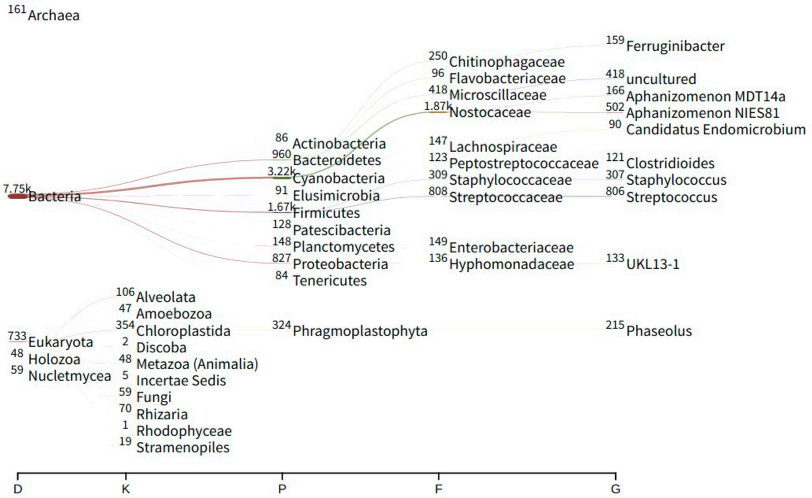

Figure S2.12: Cyanobacterial sample LMECYA 040

Supplementary Figure 2 (cont.)

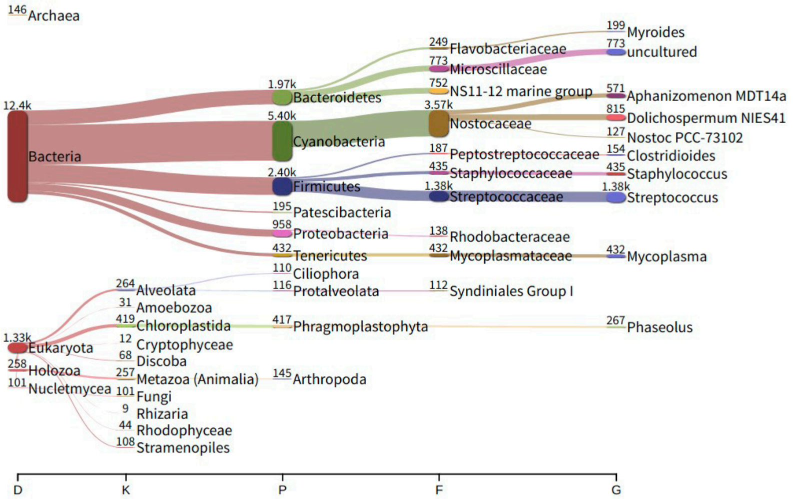

Figure S2.13: Cyanobacterial sample LMECYA 089

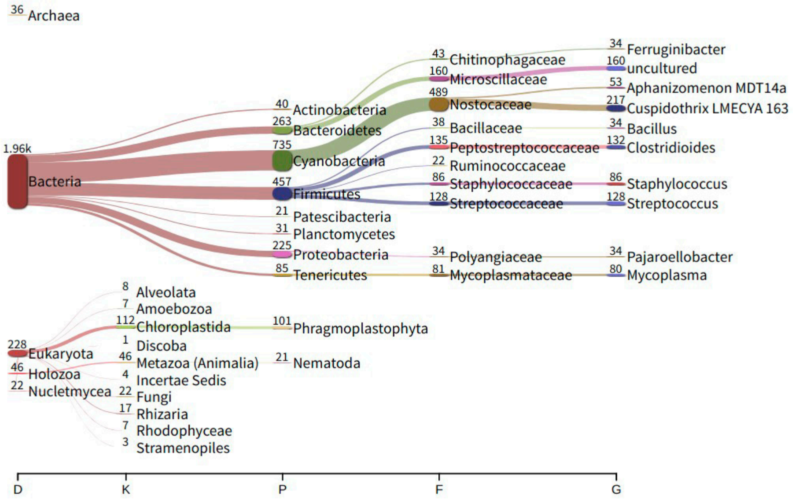

Figure S2.14: Cyanobacterial sample LMECYA 190

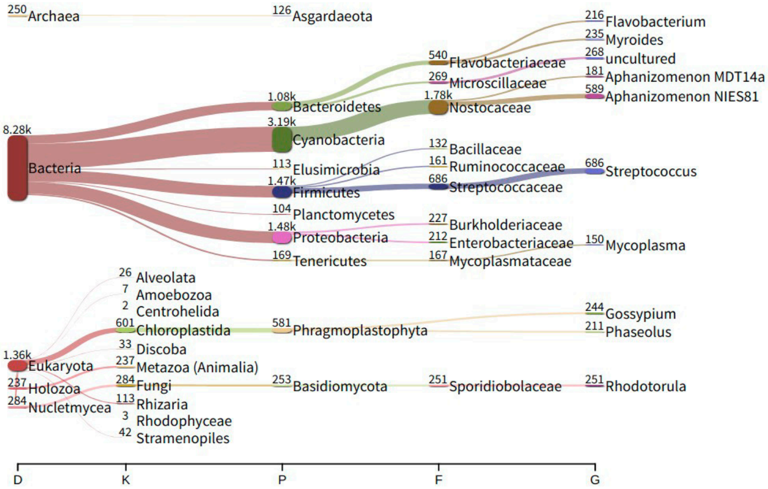

Figure S2.15: Cyanobacterial sample LMECYA 191

Supplementary Figure 2 (cont.)

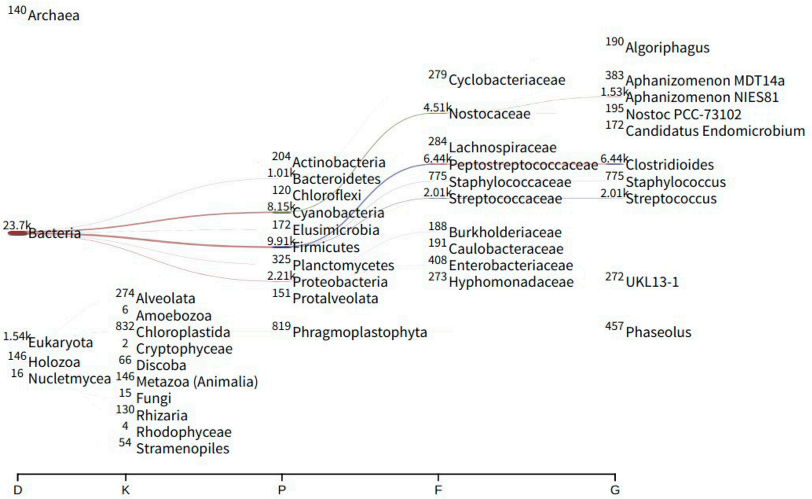

Figure S2.16: Cyanobacterial sample LMECYA 237

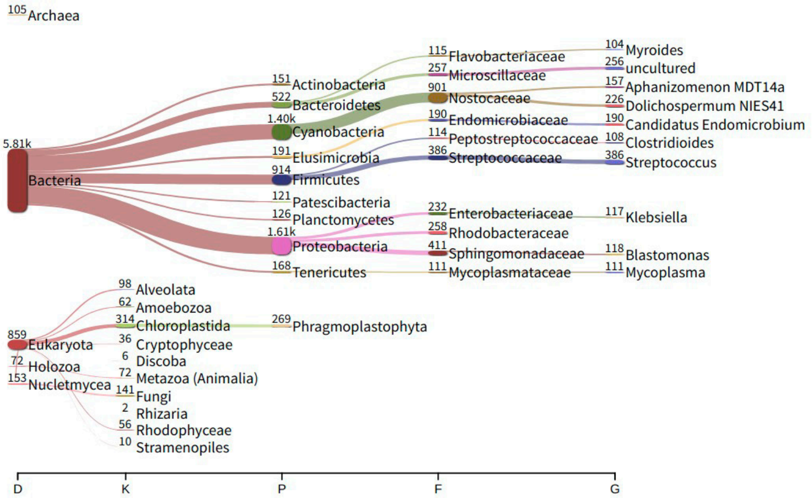

Figure S2.17: Cyanobacterial sample LMECYA 253

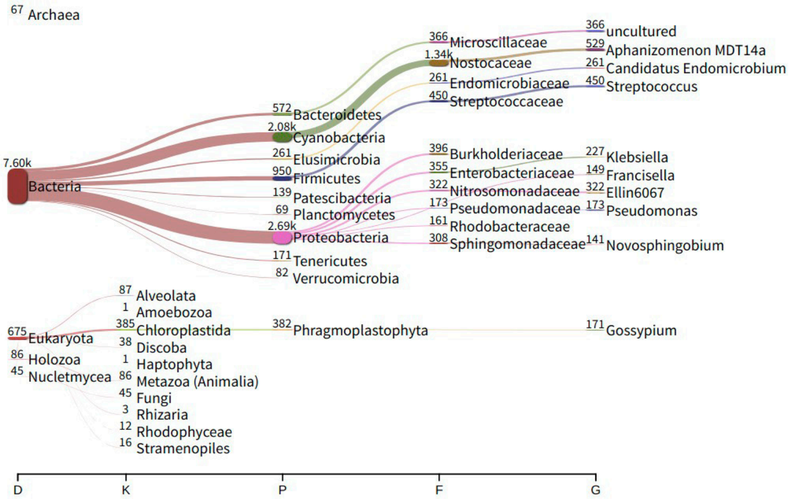

Figure S2.18: Cyanobacterial sample LMECYA 328

Supplementary Figure 2 (cont.)

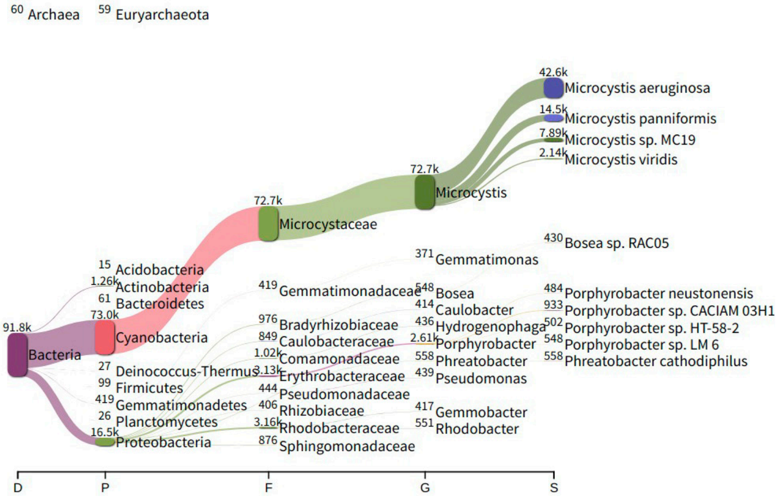

Figure S2.19: Cyanobacterial sample LMECYA 179

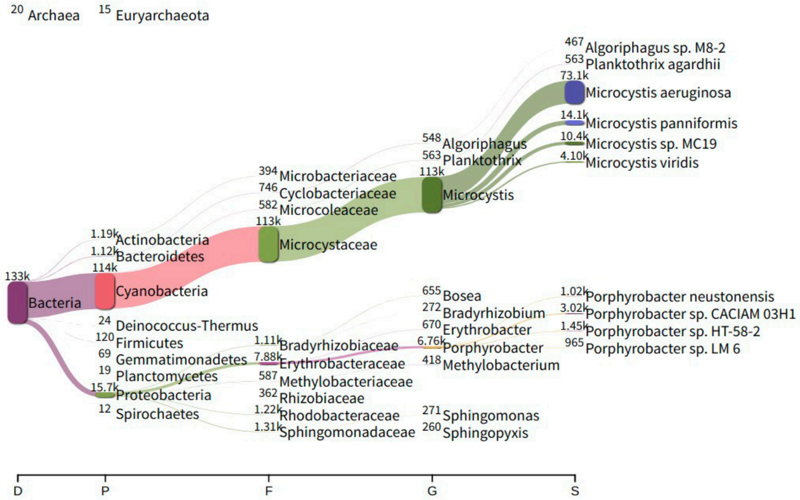

Figure S2.20: Cyanobacterial sample LMECYA 050

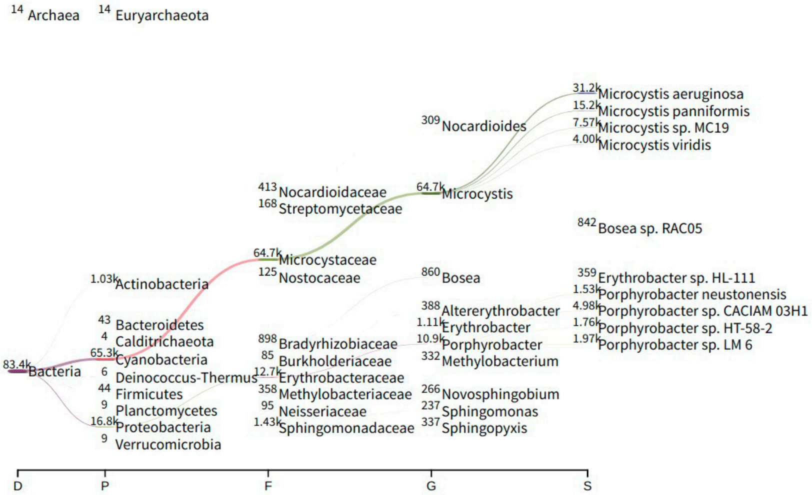

Figure S2.21: Cyanobacterial sample LMECYA 91B

Supplementary Figure 2 (cont.)

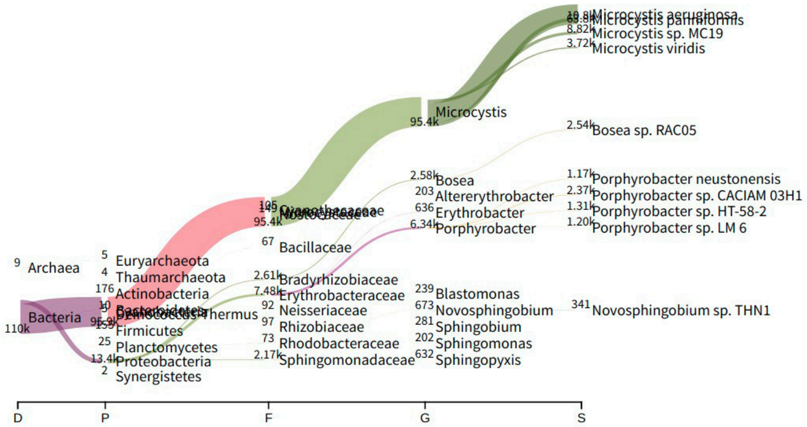

Figure S2.22: Cyanobacterial sample LMECYA 108

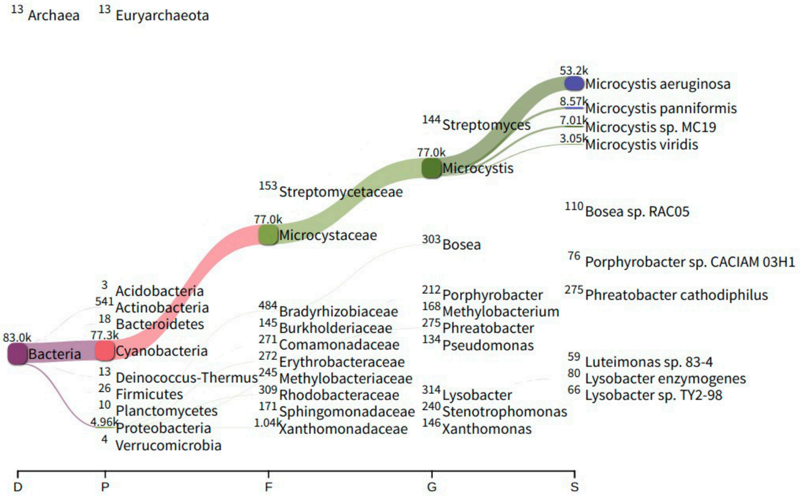

Figure S2.23: Cyanobacterial sample LMECYA 113

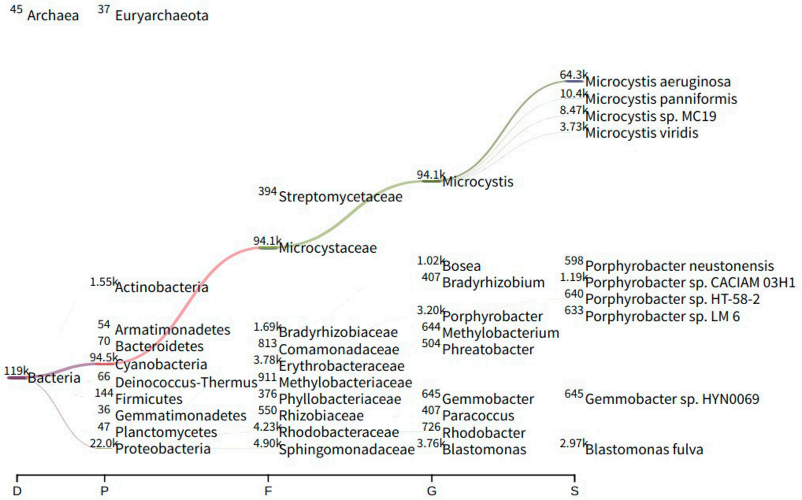

Figure S2.24: Cyanobacterial sample LMECYA 142

Supplementary Figure 2 (cont.)

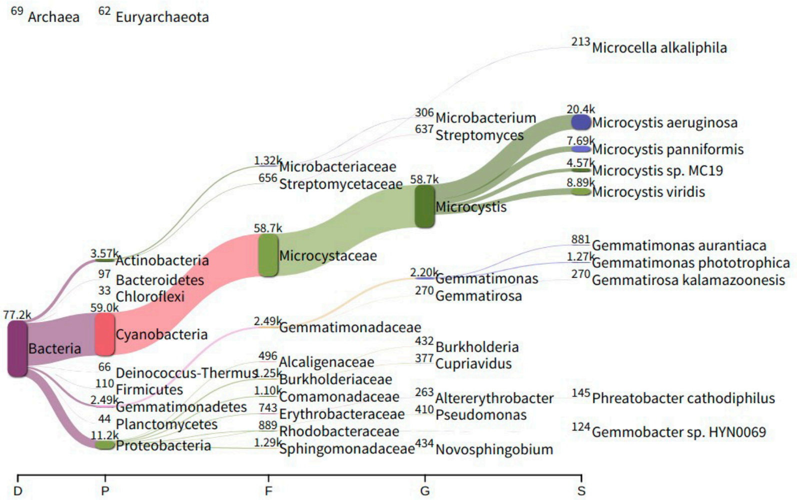

Figure S2.25: Cyanobacterial sample LMECYA 151

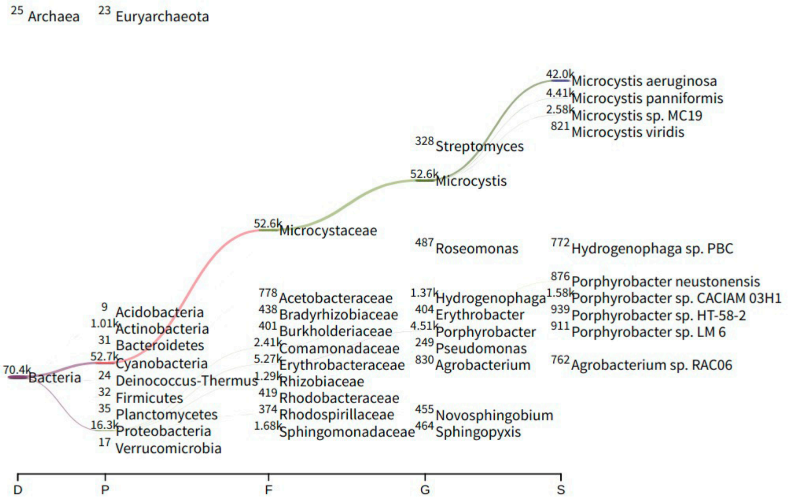

Figure S2.26: Cyanobacterial sample LMECYA 159

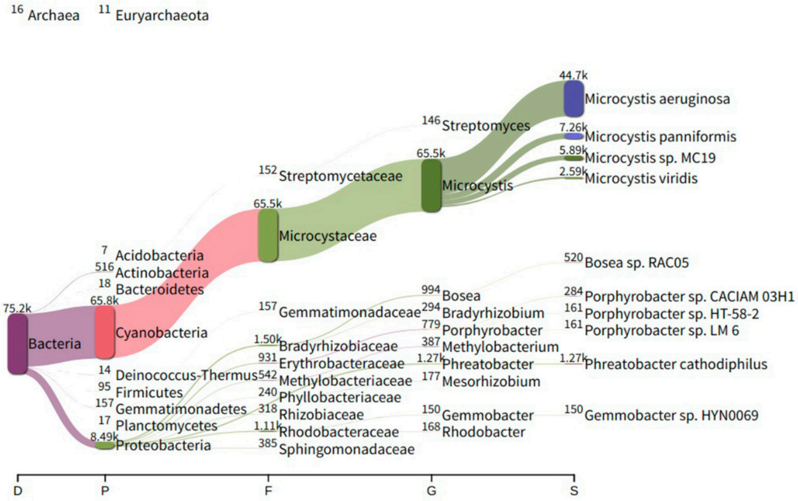

Figure S2.27: Cyanobacterial sample LMECYA 167

Supplementary Figure 2 (cont.)

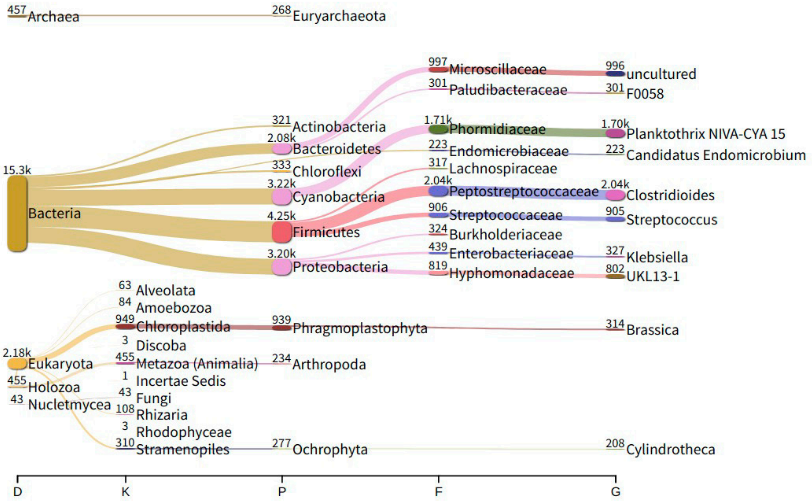

Figure S2.28: Cyanobacterial sample LMECYA 153A

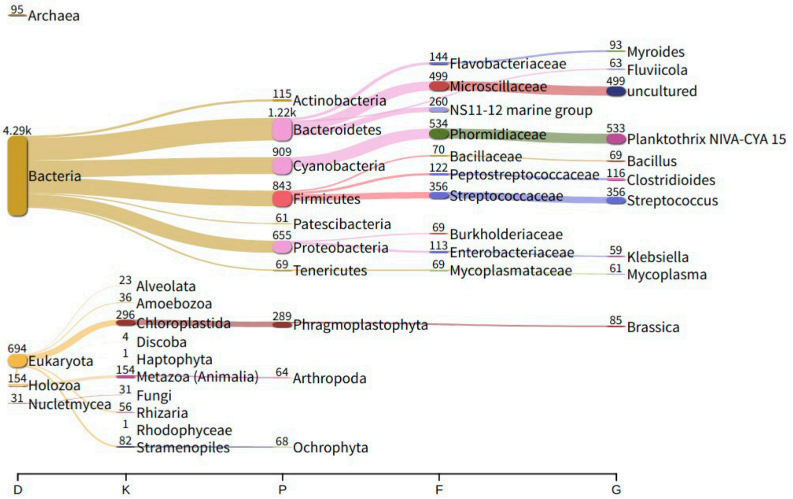

Figure S2.29: Cyanobacterial sample LMECYA 230

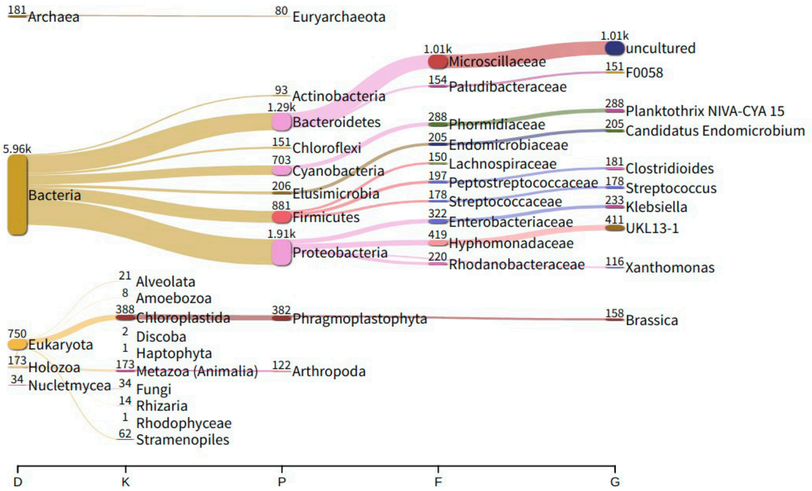

Figure S2.30: Cyanobacterial sample LMECYA 257

Supplementary Figure 2 (cont.)

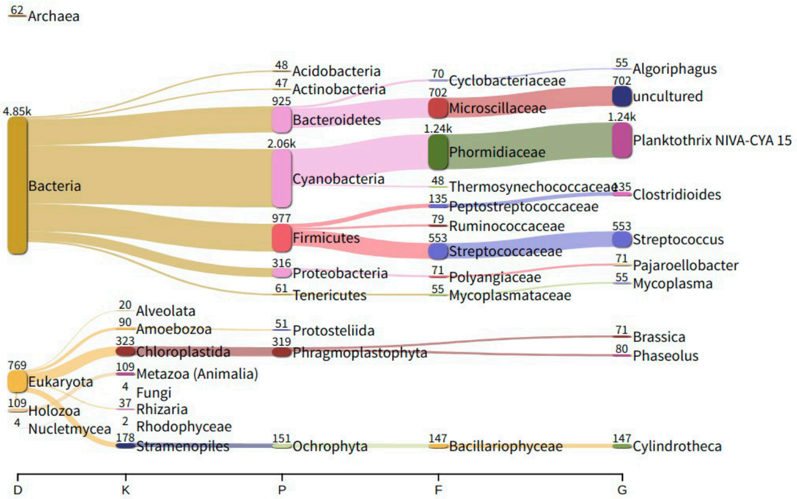

Figure S2.31: Cyanobacterial sample LMECYA 269

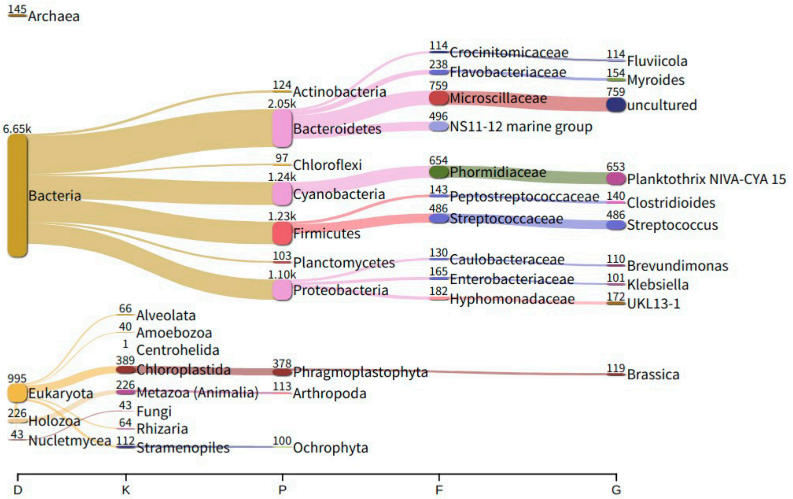

Figure S2.32: Cyanobacterial sample LMECYA 280

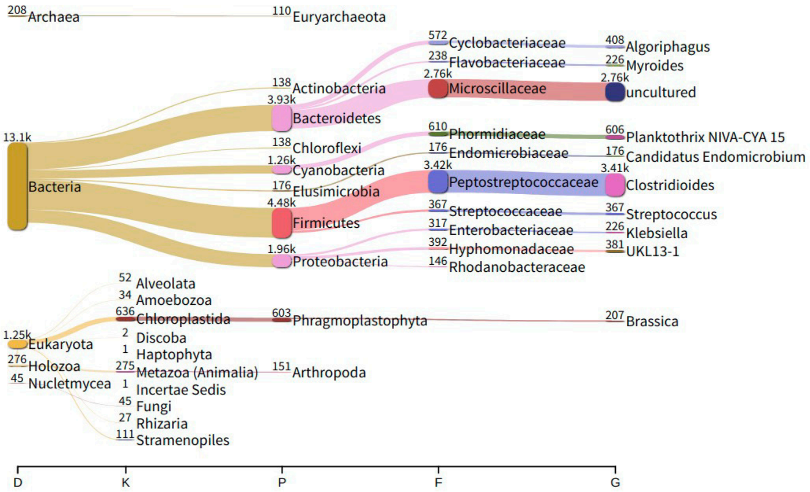

Figure S2.33: Cyanobacterial sample LMECYA 283

Supplementary Figure 2 (cont.)

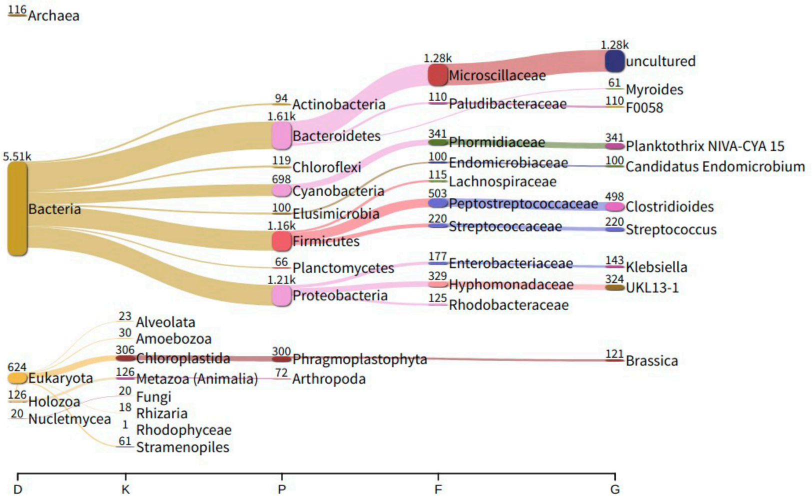

Figure S2.34: Cyanobacterial sample LMECYA 292

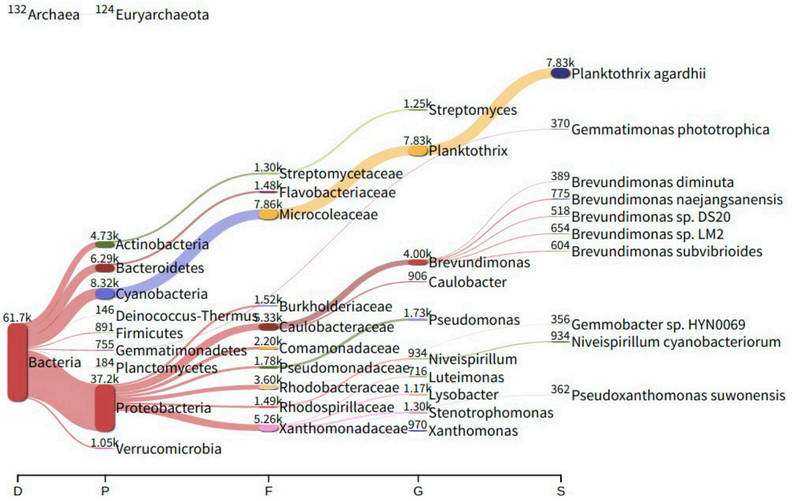

Figure S2.35: Cyanobacterial sample LMECYA 303

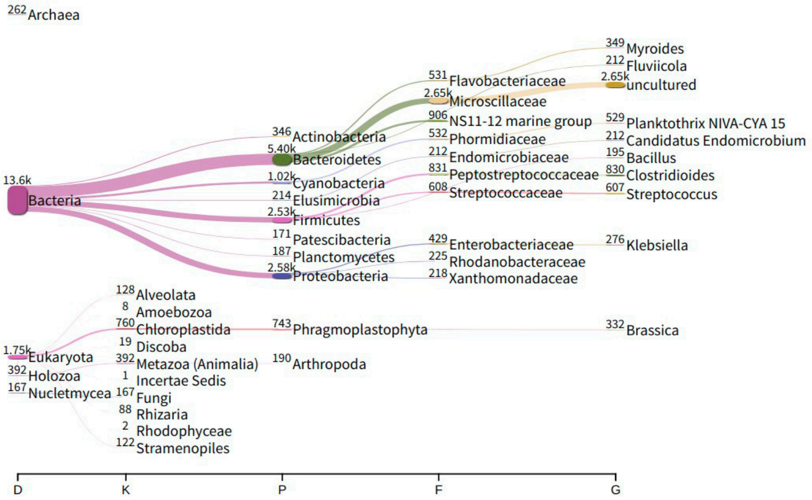

Figure S2.36: Cyanobacterial sample LEGE 06224

Supplementary Figure 2 (cont.)

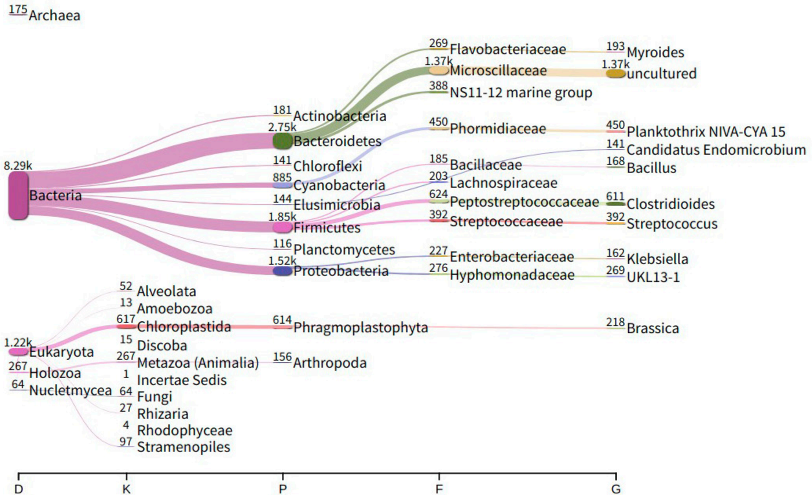

Figure S2.37: Cyanobacterial sample LEGE 06225

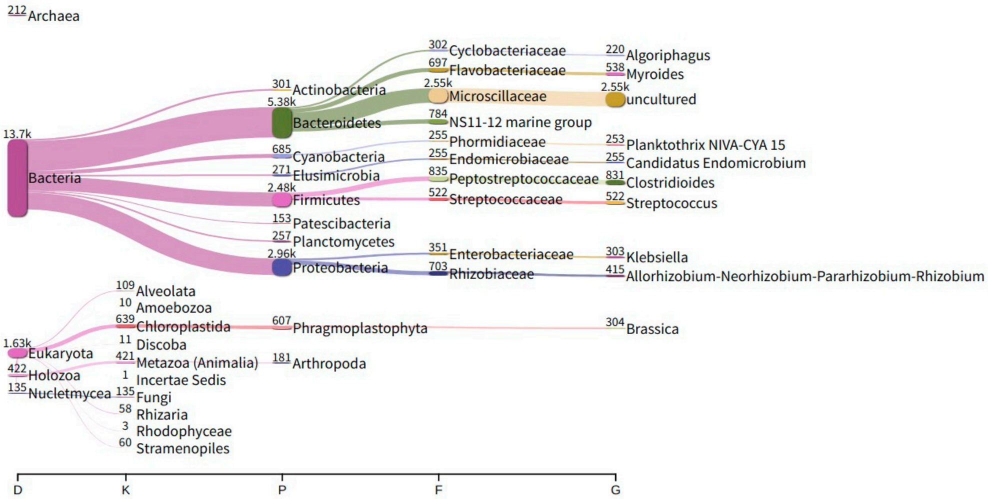

Figure S2.38: Cyanobacterial sample LEGE 06226

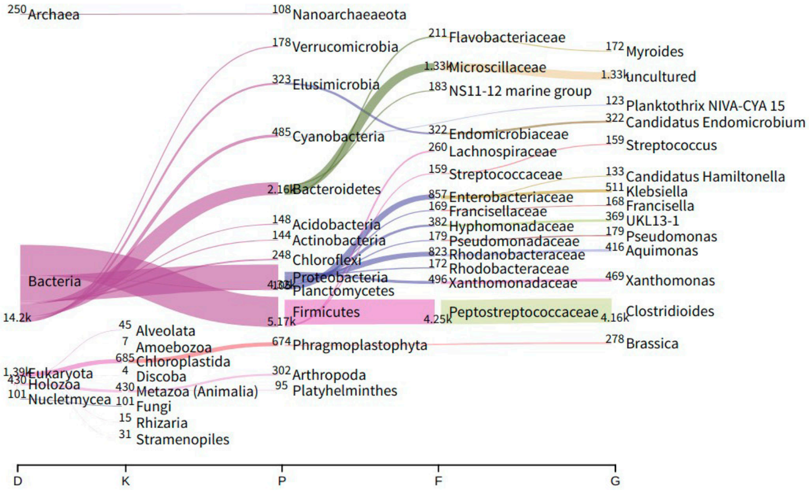

Figure S2.39: Cyanobacterial sample LEGE 06233

Supplementary Figure 2 (cont.)

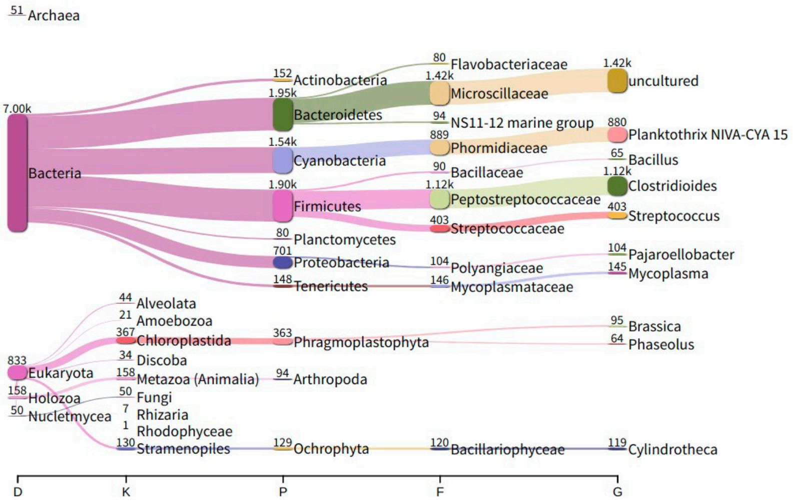

Figure S2.40: Cyanobacterial sample LEGE 07227

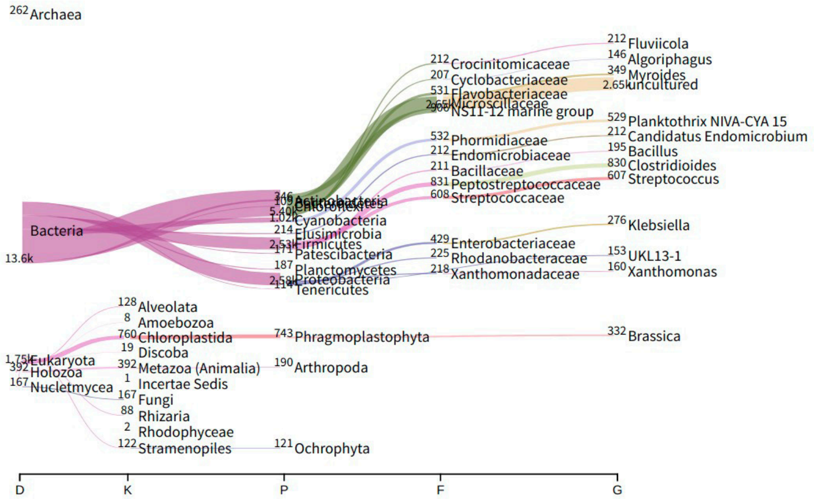

Figure S2.41: Cyanobacterial sample LEGE 07229

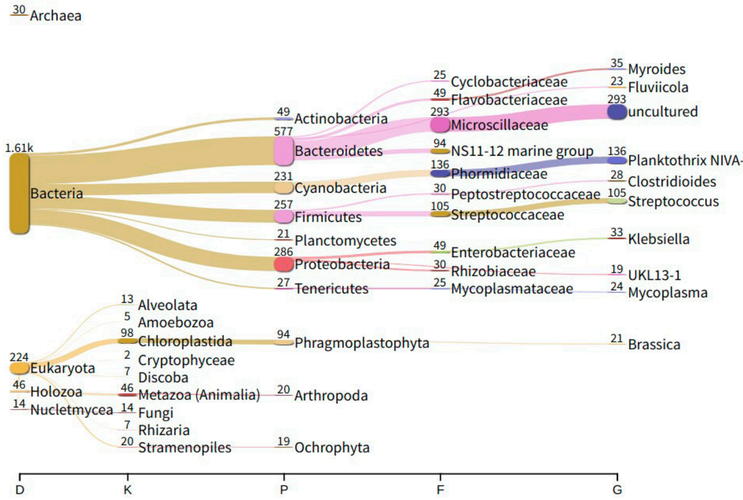

Figure S2.42: Cyanobacterial sample LEGE 07230

Supplementary Figure 2 (cont.)

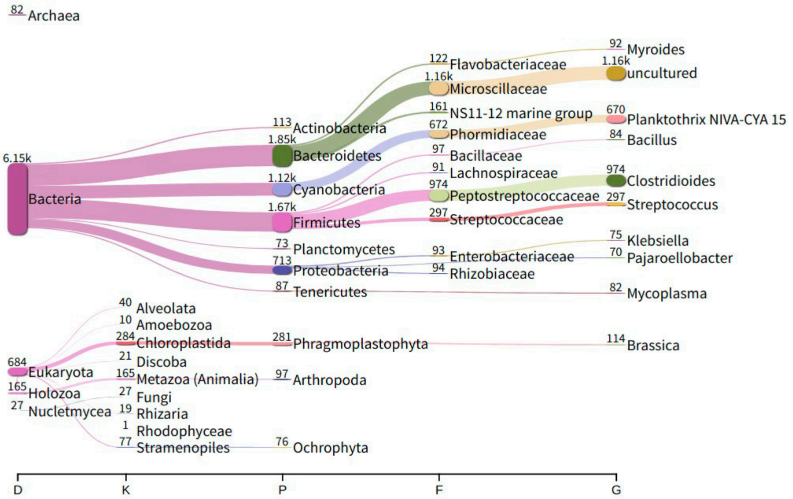

Figure S2.43: Cyanobacterial sample LEGE 07231
