# Supplementary material for: Prediction of Antibiotic Resistance Genes in Cyanobacterial Strains by Whole Genome Sequencing
Source: Microorganisms. 2025 May 28;13(6):1252. doi: 10.3390/microorganisms13061252 (PMC12195311; doi:10.3390/microorganisms13061252)
Supplement: Supplementary file 1 [file microorganisms-13-01252-s001.zip › MS 2828637 Supplementary Table 1.pdf]

Supplementary Table 1. Cyanobacteria strains' taxonomy according to a) microscopic identification (morphology); b) genomic classification using software Kraken2. The accession number of each strain is also indicated (NCBI BioProject ref. PRJNA956929); c) reclassification based on morphologic and genomic analysis, and taxonomic revisions.

**Culture collection ID***	**Morphological identification**	**Genomic analysis**		**Reclassification based on morphologic/genomic analysis and taxonomic revisions**
		**Kraken2-based identification**	**BioSample accessions**	
LMECYA 123C	*Anabaena circinale*	*Dolichospermum* sp.	SAMN34233937	*Dolichospermum circinale*
LMECYA 161	*Anabaena spiroides*	*Dolichospermum* sp.	SAMN34233938	*Dolichospermum spiroides*
LMECYA 165	*Anabaena flos-aquae*	*Sphaerospermopsis* sp.	SAMN34233939	*Sphaerospermopsis* sp.
LMECYA 178C	*Anabaena flos-aquae*	*Nodosilinea* sp.	SAMN34233940	*Nodosilinea* sp.
LMECYA 182	*Anabaena* sp.	*Sphaerospermopsis* sp.	SAMN34233941	*Sphaerospermopsis* sp.
LMECYA 204	*Anabaena* sp.	*Sphaerospermopsis* sp.	SAMN34233942	*Sphaerospermopsis* sp.
LMECYA 213	*Anabaena circinalis*	*Aphanizomenon* sp.	SAMN34233943	Dolichospermum sp.
LMECYA 246	*Anabaena bergii*	*Anabaena* sp.	SAMN34233944	*Chrysosporum bergii*
LMECYA 313	*Anabaena torulosa*	*Anabaena* sp.	SAMN34233945	*Anabaena torulosa*
LMECYA 009	*Aphanizomenon gracile*	*Aphanizomenon* sp.	SAMN34233946	*Aphanizomenon gracile*
LMECYA 031	*Aphanizomenon issastchenkoi*	*Cuspidothrix* sp.	SAMN34233947	*Cuspidothrix issastchenkoi*
LMECYA 040	*Aphanizomenon gracile*	*Aphanizomenon* sp.	SAMN34233948	*Aphanizomenon gracile*
LMECYA 089	*Aphanizomenon flos-aquae*	*Dolichospermum* sp.	SAMN34233949	*Aphanizomenon sp.*
LMECYA 190	*Aphanizomenon issastchenkoi*	*Cuspidothrix* sp.	SAMN34233950	*Cuspidothrix issastchenkoi*
LMECYA 191	*Aphanizomenon gracile*	*Aphanizomenon* sp.	SAMN34233951	*Aphanizomenon gracile*
LMECYA 237	*Aphanizomenon gracile*	*Aphanizomenon* sp.	SAMN34233952	*Aphanizomenon gracile*
LMECYA 253	*Aphanizomenon flos-aquae*	*Dolichospermum* sp.	SAMN34233953	*Aphanizomenon sp.*
LMECYA 328	*Aphanizomenon flos aquae*	*Aphanizomenon* sp.	SAMN34233954	*Aphanizomenon flos aquae*
LMECYA 179	*Microcystis aeruginosa*	*Microcystis* sp.	SAMN34233955	*Microcystis aeruginosa*
LMECYA 50	*Microcystis aeruginosa*	*Microcystis* sp.	SAMN34233956	*Microcystis aeruginosa*
LMECYA 91B	*Microcystis aeruginosa*	*Microcystis* sp.	SAMN34233957	*Microcystis aeruginosa*
LMECYA 108	*Microcystis aeruginosa*	*Microcystis* sp.	SAMN34233958	*Microcystis aeruginosa*
LMECYA 113	*Microcystis aeruginosa*	*Microcystis* sp.	SAMN34233959	*Microcystis aeruginosa*
LMECYA 142	*Microcystis aeruginosa*	*Microcystis* sp.	SAMN34233960	*Microcystis aeruginosa*
LMECYA 151	*Microcystis aeruginosa*	*Microcystis* sp.	SAMN34233961	*Microcystis aeruginosa*
LMECYA 159	*Microcystis aeruginosa*	*Microcystis* sp.	SAMN34233962	*Microcystis aeruginosa*
LMECYA 167	*Microcystis aeruginosa*	*Microcystis* sp.	SAMN34233963	*Microcystis aeruginosa*
LMECYA 153A	*Planktothrix agardhii*	*Planktothrix* sp.	SAMN34233964	*Planktothrix agardhii*
LMECYA 230	*Planktothrix agardhii*	*Planktothrix* sp.	SAMN34233965	*Planktothrix agardhii*
LMECYA 257	*Planktothrix agardhii*	*Planktothrix* sp.	SAMN34233966	*Planktothrix agardhii*
LMECYA 269	*Planktothrix agardhii*	*Planktothrix* sp.	SAMN34233967	*Planktothrix agardhii*
LMECYA 280	*Planktothrix agardhii*	*Planktothrix* sp.	SAMN34233968	*Planktothrix agardhii*
LMECYA 283	*Planktothrix agardhii*	*Planktothrix* sp.	SAMN34233969	*Planktothrix agardhii*
LMECYA 292	*Planktothrix agardhii*	*Planktothrix* sp.	SAMN34233970	*Planktothrix agardhii*
LMECYA 303	*Planktothrix agardhii*	*Planktothrix* sp.	SAMN34233971	*Planktothrix agardhii*
LEGE 06224	*Planktothrix mougeotii*	*Planktothrix* sp.	SAMN34233972	*Planktothrix mougeotii*
LEGE 06225	*Planktothrix mougeotii*	*Planktothrix* sp.	SAMN34233973	*Planktothrix mougeotii*
LEGE 06226	*Planktothrix mougeotii*	*Planktothrix* sp.	SAMN34233974	*Planktothrix mougeotii*
LEGE 06233	*Planktothrix mougeotii*	*Planktothrix* sp.	SAMN34233975	*Planktothrix mougeotii*
LEGE 07227	*Planktothrix mougeotii*	*Planktothrix* sp.	SAMN34233976	*Planktothrix mougeotii*
LEGE 07229	*Planktothrix mougeotii*	*Planktothrix* sp.	SAMN34233977	*Planktothrix mougeotii*
LEGE 07230	*Planktothrix mougeotii*	*Planktothrix* sp.	SAMN34233978	*Planktothrix mougeotii*
LEGE 07231	*Planktothrix mougeotii*	*Planktothrix* sp.	SAMN34233979	*Planktothrix mougeotii*
