# Supplementary material for: Prediction of Antibiotic Resistance Genes in Cyanobacterial Strains by Whole Genome Sequencing
Source: Microorganisms. 2025 May 28;13(6):1252. doi: 10.3390/microorganisms13061252 (PMC12195311; doi:10.3390/microorganisms13061252)
Supplement: Supplementary file 1 [file microorganisms-13-01252-s001.zip › MS 2828637 Supplementary Table 2.pdf]

Supplementary Table 2. Percentage of classified cyanobacterial metagenome reads that present the best alignment with each genus. Based on the reads that match the reference 16S sequences belonging to said genus. Intensity of cell coloring is related to the proportion of reads on a sample that are attributed to a given genus. Higher color intensity denotes highest correspondences.

Table S2.1: *Anabaena* samples

Name	Rank	TID	Max	LMECYA 123C	LMECYA 161	LMECYA 165	LMECYA 178C	LMECYA 182	LMECYA 204	LMECYA 213	LMECYA 246	LMECYA 313
Dolichospermum NIES41	G	25366	85.36	78.58%	85.36%	0.5282%		0.451%	0.5942%	3.597%	0.5535%	1.469%
Sphaerospermopsis BCCUSP55	G	25397	75.4			71.24%	0.1063%	55.02%	75.4%	0.3597%	0.09225%	10.16%
Aphanizomenon NIES81	G	25346	70.14	4.184%	3.214%	13.62%		14.71%	10.52%	70.14%		23.75%
Anabaena PMC215.03	G	25342	65.5			0.05869%					65.5%	
Nodosilinea PCC-7104	G	25490	62.31	0.2128%			62.31%					
Anabaena HBU1	G	25339	21.91									21.91%

Table S2.2: *Aphanizomenon* samples

Name	Rank	TID	Max	LMECYA 009	LMECYA 031	LMECYA 040	LMECYA 089	LMECYA 190	LMECYA 191	LMECYA 234	LMECYA 253	LMECYA 328
Aphanizomenon MDT14a	G	25345	73.17	24.81%	17.22%	16.9%	32.31%	12.71%	14.96%	14.18%	31.46%	73.17%
Aphanizomenon NIES81	G	25346	56.65	37.36%	8.278%	51.12%	2.377%	4.796%	48.68%	56.65%	2.806%	0.9682%
Cuspidothrix LMECYA 163	G	25356	52.04		48.54%	0.8147%	0.1132%	52.04%	0.1653%	0.2592%		0.4149%
Dolichospermum NIES41	G	25366	46.12	3.396%	0.7526%	4.073%	46.12%	5.276%	11.24%	2.592%	45.29%	6.501%
Thermosynechococcus BP-1	G	25533	11.23	11.23%	2.446%	4.379%	0.9055%	3.357%	3.636%	4.184%	0.2004%	0.2766%
Nostoc PCC-73102	G	25384	10.22	5.66%	9.501%	7.434%	7.187%	3.837%	5.041%	7.22%	10.22%	8.299%

Table S2.3: *Microcystis* samples

Name	Rank	TID	Max	LMECYA 179	LMECYA 050	LMECYA 91B	LMECYA 108	LMECYA 113	LMECYA 142	LMECYA 151	LMECYA 159	LMECYA 167
Microcystis PCC-7914	G	25326	69.47	55.88%	57.63%	50.91%	63.51%	46.84%	69.47%	54.55%	56.9%	58.9%
Crocosphaera WH 0003 (UCYN-B)	G	25320	14.71	14.71%	9.322%	7.273%	10.81%	7.595%	6.316%	4.545%	1.724%	8.219%
Snowella 0TU37S04	G	25329	1.471	1.471%								
Nostoc PCC-73102	G	25384	29.11	16.18%	17.8%	20%	24.32%	29.11%	12.63%	22.73%	25.86%	20.55%
Aphanizomenon NIES81	G	25346	1.471	1.471%								
Petalonema ANT.LG2.8	G	25388	1.471	1.471%								

Table S2.4: *Planktothrix agardhii* samples

Name	Rank	TID	Max	LMECYA 153A	LMECYA 230	LMECYA 257	LMECYA 269	LMECYA 280	LMECYA 283	LMECYA 292	LMECYA 303
Planktothrix NIVA-CYA 15	G	25440	90.55	86.06%	86.81%	88.62%	90.55%	85.92%	86.7%	87.89%	
Trichodesmium IMS101	G	25442	0.05051	0.05051%							
Desmonostoc PCC-7422	G	25365	4.343	4.343%	3.42%	3.385%	3.077%	3.816%	3.72%	2.32%	
Tolypothrix PCC-7601	G	25402	0.1431	0.05051%					0.1431%		
Rivularia PCC-7116	G	25391	0.7153	0.05051%				0.2632%	0.7153%	0.2577%	
Aphanizomenon NIES81	G	25346	0.05051	0.05051%							

Table S2.5: *Planktothrix mougeotii* samples

Name	Rank	TID	Max	LEGE 06224	LEGE 06225	LEGE 06226	LEGE 06233	LEGE 07227	LEGE 07229	LEGE 07230	LEGE 07231
Planktothrix NIVA-CYA 15	G	25440	86.08	80.03%	82.87%	73.12%	82.55%	74.75%	82.24%	86.08%	83.85%
Desmonostoc PCC-7422	G	25365	8.081	3.782%	2.762%	3.757%	4.027%	8.081%	4.299%	3.165%	3.004%
Thermosynechococcus BP-1	G	25533	8.081	4.992%	5.157%	6.358%	4.027%	8.081%	4.486%	5.696%	4.631%
uncultured	G	25460	7.225	0.3026%	1.105%	7.225%	1.342%		0.3738%		0.1252%
Dapisostemonum CCIBt 3536	G	25283	5.051	3.782%	3.131%	3.468%	2.013%	5.051%	5.047%	1.899%	4.38%
Phormidium SAG 81.79	G	25412	4.539	4.539%	3.315%	2.312%	2.685%	1.01%	2.991%	3.165%	3.379%
