# Supplementary material for: Prediction of Antibiotic Resistance Genes in Cyanobacterial Strains by Whole Genome Sequencing
Source: Microorganisms. 2025 May 28;13(6):1252. doi: 10.3390/microorganisms13061252 (PMC12195311; doi:10.3390/microorganisms13061252)
Supplement: Supplementary file 1 [file microorganisms-13-01252-s001.zip › MS 2828637 Supplementary Table 4.pdf]

Supplementary Table 4: Per base genome coverage of recovered cyanobacteria genomes as the average number of reads displaying information about each nucleotide.

Sample ID	CB coverage (x)
LMECYA 123C	58.21
LMECYA 161	8.95
LMECYA 165	65.44
LMECYA 178C	94.49
LMECYA 182	75.44
LMECYA 204	70.83
LMECYA 213	26.40
LMECYA 246	60.02
LMECYA 313	49.28
LMECYA 009	41.77
LMECYA 031	40.36
LMECYA 040	42.13
LMECYA 089	68.31
LMECYA 190	14.75
LMECYA 191	42.14
LMECYA 237	149.62
LMECYA 253	20.04
LMECYA 328	28.08
LMECYA 179	7.07
LMECYA 50	8.86
LMECYA 91B	5.78
LMECYA 108	9.09
LMECYA 113	6.89
LMECYA 142	8.65
LMECYA 151	5.30
LMECYA 159	5.21
LMECYA 167	6.12
LMECYA 153A	64.70
LMECYA 230	26.54
LMECYA 257	13.87
LMECYA 269	54.94
LMECYA 280	30.81
LMECYA 283	25.89
LMECYA 292	15.40
LMECYA 303	---
LEGE 06224	21.33
LEGE 06225	18.82
LEGE 06226	11.60
LEGE 06233	6.61
LEGE 07227	---
LEGE 07229	36.41
LEGE 07230	6.23
LEGE 07231	23.82
